# How does an intervention work?—English Version: Development of an effect model for a complex intervention to prevent recurring or persistent pain using the example of PAIN 2.0

**DOI:** 10.1007/s00482-024-00860-8

**Published:** 2025-01-21

**Authors:** Ulrike Kaiser, Leonie Schouten, Greta Hoffmann, Anke Preißler, Franziska Adler, Louise Zinndorf, Anne Kästner, Beatrice Metz-Oster, Enya Höffner, Gabriele Lindena, PAIN 2.0-Team, Thomas Isenberg, André Möller, Felix Rottke, Anja Waidner, Jakob Seitz, Ulrike Kaiser, Anne Gärtner, Anke Preißler, Greta Hoffmann, Julia Pritzke Michael, Frank Petzke, Leonie Schouten, Franziska Adler, Christian Geber, Beatrice Metz-Oster, Lena Milch, Louise Zinndorf, Enya Höffner, Julia Ruff, Thomas Kohlmann, Sandra Meyer-Moock, Daniel Szczotkowski, Ursula Marschall, Catharina Schumacher, Thomas Kohlmann, Sandra Meyer-Moock, Daniel Szczotkowski, Christian Geber, Frank Petzke, Lena Milch, Anne Gärtner

**Affiliations:** 1 University Hospital Schleswig-Holstein/Lübeck, Lübeck, Germany; 2 Pain Medicine, Department of Anesthesiology, University Medical Center Göttingen, Robert-Koch-Straße 40, 37075 Göttingen, Germany; 3 University Pain Center, University Hospital Carl Gustav Carus, Dresden, Germany; 4 German Red Cross Pain Center Mainz, Mainz, Germany; 5 German Pain Society, Berlin, Germany; 6 Institute for Community Medicine, University Medicine Greifswald, Greifswald, Germany

**Keywords:** Program theory, Theory of action, Secondary prevention of chronic pain, Complex intervention, Interdisciplinary pain management program, Programmtheorie, Wirkmodell, Sekundäre Prävention chronischer Schmerzen, Komplexe Intervention, Interdisziplinäre multimodale Schmerztherapie

## Abstract

In addition to the usual evaluation approach (usually a clinical randomized trial in the sense of the question: does an intervention work), complex interventions require further systematic investigations to prove their effectiveness. The role of the context in which the intervention is delivered is essential here, as is consideration of the question of *why* an intervention works (or does not work). Detailed recommendations exist for the planning and implementation of effectiveness studies on complex interventions, to which interdisciplinary multimodal pain therapy undoubtedly belongs. In an effectiveness model, concrete, verifiable assumptions are formulated as to how an intervention produces changes that are reflected in the endpoint. This article provides a brief introduction to methodological approaches to effectiveness research on complex interventions and uses the PAIN 2.0 project (01NVF20023) to describe in concrete terms what an effectiveness model for interdisciplinary multimodal pain therapy for the prevention of chronic pain in an outpatient setting might look like.

**Supplementary Information:**

The online version of this article (10.1007/s00482-024-00860-8) includes supplementary information on process and control variables.

## Abstract (de)

Komplexe Interventionen bedürfen für den Nachweis ihrer Wirksamkeit neben dem gängigen Evaluationsansatz (meist eine klinisch-randomisierte Studie i. S. der Frage: WIRKT eine Intervention?) weiterer systematischer Untersuchungen. Wesentlich sind hier die Rolle des Kontextes, in dem die Intervention vermittelt wird, sowie die Berücksichtigung der Frage, WARUM eine Intervention wirkt (bzw. nicht wirkt). Für die Planung und Durchführung von Effektivitätsstudien zu komplexen Interventionen, zu denen die interdisziplinäre multimodale Schmerztherapie unzweifelhaft gehört, existieren ausführliche Empfehlungen. In einem Wirkmodell werden konkrete, überprüfbare Annahmen dazu formuliert, wodurch eine Intervention Veränderungen erzeugt, die sich im Endpunkt widerspiegeln. In diesem Artikel wird eine kurze Einführung zu methodischen Ansätzen der Effektivitätsforschung zu komplexen Interventionen gegeben und anhand des Projektes PAIN 2.0 (01NVF20023) konkret beschrieben, wie ein Wirkmodell für eine interdisziplinäre multimodale Schmerztherapie zur Prävention von chronischen Schmerzen in einem ambulanten Setting aussehen kann.

## Introduction

With the implementation of the Innovation Fund in 2016, the Federal Joint Committee is promoting new, innovative forms of care and complex interventions. The associated aim is to improve the quality and efficiency of care across sectors by eliminating care deficits and optimizing cooperation within and between different care areas, care facilities, and professional groups [4].

A large proportion of the projects in the German Innovation Fund are currently being carried out in the sense of an effectiveness-oriented approach: the focus of these studies is on the question of *whether* an intervention is effective in the care system under everyday conditions [23]. In particular, the evaluation is intended to provide decision-making aids for implementation in standard care, as only positively evaluated care innovations are transferred to standard care in the long term [4].

Two projects of the German Pain Society, BARMER and the University Medicine Greifswald pursue the goal of offering early interdisciplinary care based on a biopsychosocial model to patients with recurring or persistent pain and the risk of chronification. The primary aim is to prevent (progressing) pain chronification through early interdisciplinary diagnosis and treatment.

The PAIN2020 project (Patientenorientiert.Abgestuft.Interdisziplinär.Netzwerk; 01NVF17049, runtime 2018–2022) developed and evaluated early interdisciplinary multimodal pain diagnostics for this target group [26]. In addition, two low-threshold, outpatient, interdisciplinary therapy services were developed and piloted [26]. This experience gave rise to the follow-up project PAIN 2.0 (Patientenorientiert.Abgestuft.Interdisziplinär.Netzwerk—Therapie; 01NVF20023, runtime 2022–2025), in which outpatient interdisciplinary multimodal pain therapy (A-IMPT) is to be evaluated in terms of secondary prevention.

### Theory-based interventions in health services research

It can be assumed that the majority of interventions carried out in health services research must be categorized as complex interventions. We speak of complex interventions when these interventions consist of several components, address different behaviors, require different skills and expertise from the health care provider or require a certain flexibility of the intervention (e.g., in terms of patient orientation) or its components, respectively [41]. The role of the context contributes greatly to the complexity of the intervention and its effectiveness [40].

One major aim of health services research is to develop ready-to-use interventions that can be applied in standard care under given everyday conditions for practitioners and are useful for patients. With regard to complex interventions, however, the question of *WHETHER* an intervention is effective cannot be sufficient. In the sense of a learning care system, the aim must be to understand for whom an intervention is useful under which conditions and *WHY* it works (or does not work) [41].

#### Concept development for complex interventions

Various recommendations now exist for the sound development of new interventions [12]. Consensus is that existing evidence, current or newly developed theories, involvement of diverse stakeholders, piloting, and some form of evidence testing should be applied as part of a development process. An intervention should build on existing gaps or deficits in care, be evidence-based, and have a strong theory base. It should connect to the needs, abilities, and preferences of the target group on the one hand and the regional availability within the healthcare system on the other [3].

The Medical Research Council (MRC, [41]) recommends an iterative process for the development of new or adaptation of existing interventions, consisting of the following steps: development, feasibility analysis, evaluation, and implementation of the new form of care.

After describing the existing issues, current evidence is identified with regard to the description of the target group’s needs, relevant mechanisms of action and previously investigated interventions [3, 41]. To ensure a sufficient theoretical basis, existing theories can be identified or developed (examples include program theory, theory of change, etc.). The planning of the procedure or process should take into account the later context of the intervention, including the identification of resources and barriers. Based on this, both the process and outcome parameters as well as the subsequent design for the evaluation are customized [3, 41]. As part of the feasibility analysis, barriers and prerequisites as well as the feasibility of the intervention are examined in the form of a pilot before it is then evaluated in terms of its effectiveness using a suitable methodology. After appropriate adjustments and further developments from the previous steps (in particular the feasibility and effectiveness analyses), it is possible to transfer the intervention into the health care system, which in turn should also be accompanied by an evaluation (in the form of an implementation study; [41]).

#### Concept and significance of program theories (‘effect models’) for testing complex interventions

To design the intervention as such, so-called program theories (or effect models) are developed on the basis of theoretical assumptions and existing evidence. These are based on research into current scientific findings (including those outside the own specialist area), knowledge of regional health-related conditions and an explicit formulation of mechanisms of action (desired and undesired) [4, 41]. In concrete terms, both the targeted intervention-specific effects as well as expected side effects and impacts are formulated. Moderating or mediating relationships between the variables should be taken into account. Temporal aspects such as the timing of the intervention, its duration and the sustainability of the effects are depicted in the model. In addition to the processes on the basis of which the effectiveness of the intervention is assumed (mechanisms of action), the effect model can also describe how the skills and abilities imparted can be expected to be transferred to the recipients’ everyday lives (Dorsch Lexikon der Psychologie, access 16 April 2024: https://dorsch.hogrefe.com/stichwort/programmtheorie#search=299aa7cb65cad761c536fa2827f31ef1&offset=1). A comprehensive program theory, thus, enables the identification and operationalization of relevant variables and the formulation of concrete hypotheses for the empirical testing of the intervention and its effect in the context of an evaluation study.

Program theory focuses on the effects within the intervention and their interaction with the context. It distinguishes between the Theory of Action and the Theory of Change. The Theory of Action includes conditions and activities for the effect of a measure, which should be considered and planned as part of the establishment of the program or intervention with regard to its implementation. The Theory of Change, on the other hand, contains the (causal) assumptions regarding the specific mode of action of the interventions and their relationship to the desired, targeted outcome or result. It is understood as an approach to describe how a program achieves certain long-term results through a logical sequence of intermediate outcomes [46]. The development of program theories is method-neutral [10]. In addition to evidence synthesis, participatory elements (e.g., focus groups with practitioners and patients alike) and qualitative methods are used to develop the concept.

### Background to the new health care approach of outpatient interdisciplinary multimodal pain therapy

The concept of pain is based on the interaction of biological factors with intrapersonal factors (such as mood, behavior, cognition) and interpersonal–social constellations (such as workplace, relationship environment, biographical imprint) [37]. In addition, use of diagnostic procedures and conveyance of therapeutic concepts in the healthcare system can also contribute to chronification. It can be assumed that one-sided somatic therapy approaches, somatically oriented overdiagnosis, and inappropriate use of medication lead to an overvaluation of somatic findings and to a predominant somatic disease model on the part of those affected.

For PAIN 2.0, the risk factors listed in Table 1 were considered, as they have already been included in guidelines recommendations [7].

**Table 1:** Risk factors considered in the project PAIN 2.0

*Somatic*	Current incapacity to work for 4 weeks or cumulative incapacity to work of at least 6 weeks in the past 12 months
	Increasingly spreading pain in terms of the location
	Indications of a somatization tendency
*Psychological–cognitive behavioral*	Pronounced (verbal/nonverbal) pain behavior
	Pain-promoting pain processing (focusing, fears, catastrophizing)
	*Pain-promoting, disease-maintaining behaviors* – Protective and avoidance behavior – Excessive demands, task persistence (excessive endurance) – High health care utilization, demand for continued sick leave or continued diagnostics
*Psychological–affective*	Depressive symptoms in experience and/or behavior
	State of mind characterized by anxiety, anger, or increased stress
*Social*	Indications of increased stress in the social environment (job, family, partnership, etc.)

Against the background of these scientific findings, the question arises as to how secondary prevention (in the sense of preventing the onset of a manifest chronic pain disorder [32]) can be achieved. In 2020, the International Association for the Study of Pain (IASP) launched the Global Year for the prevention of pain to raise awareness of this field and calls were made for increased development and use of secondary prevention programs [15, 18]. However, the respective available evidence is still quite limited. Single projects on the secondary prevention of pain in the outpatient sector (mostly site-specific, not nationwide) show that improvements or even the absence of chronification were achieved in patients with pain [2, 13] or that therapy approaches based on risk profiles were resource-saving and successful [24]. Based on initial systematic reviews (mainly for musculoskeletal pain), a combination of education (imparting knowledge about the multicausal genesis of pain) and guided physical exercises for short- and medium-term periods is recommended for interventions focusing on secondary prevention [30, 43].

### Theory of Change for interdisciplinary pain management—an example

The A‑IMPT is understood as a complex intervention. Experience from the PAIN2020 project indicates that even characteristics of the treatment or the settings (e.g., procedures, staff qualifications) appear to have a direct impact on the outcome of an intervention [25]. In line with available recommendations, it was therefore important to expand the effectiveness evaluation of PAIN 2.0 with an effect model (in the sense of a theory of change) to be able to explain both its effects and the differences potentially caused by context more precisely.

#### Objective of the new health care concept A-IMPT

The PAIN 2.0 project aims to develop an outpatient, i.e., low-threshold intervention (secondary prevention) for patients with recurring or persistent pain and a risk of chronification. It is intended to address the needs of those affected as well as the practicability in the existing health care system and prevent (progression of) chronification.

#### Target population for A-IMPT

The new form of care is aimed at patients with pain that has occurred for the first time (at least 6 weeks ago) or has been recurring or ongoing for a longer time. They experience relevant, pain-related impairments (like sick-leave, everyday life/family/leisure, and household tasks/work) associated with a decreased quality of life. They are at risk of chronification due to accompanying biopsychosocial factors (Table 1).

#### Setting for A-IMPT

Based on the biopsychosocial concept of the experience of pain [37] the new form of care should be developed for an interdisciplinary treatment setting, use multimodal interventions, and be implementable in an outpatient setting.

In accordance with the chosen conceptual framework, basic recommendations of the professional associations for ensuring interdisciplinarity are applied [1, 31]. Core criteria for high-quality interdisciplinary multimodal pain therapy (IMPT, [1]) are

- Multiprofessionalism (at least two specialist disciplines),
- Consistently integrated teamwork (via regular team meetings and rounds),
- The use of evidence-based approaches as part of IMST and
- A structured approach (including the consistent documentation of treatment and the implementation of quality assurance through the use of standardized questionnaires).

#### Development of the new form of A-IMPT care

The development of the pilot form of the new form of care has been reported in detail [36].

#### Brief description of the new form of A-IMPT care

A more detailed description of the theories and concepts used to develop the new form of care is in preparation.

**Fig. 1:**
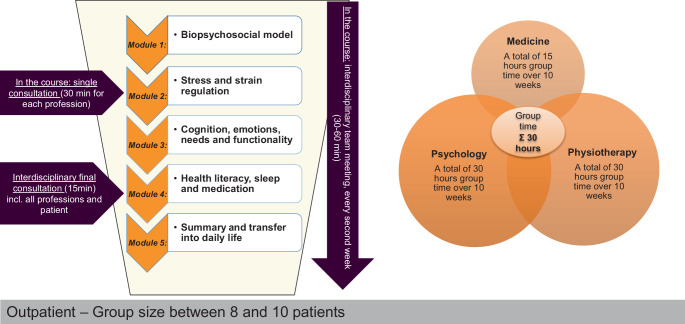
Schedule and content of A‑IMPT

The intervention is designed as a group intervention for closed groups of 8–10 participants (see also [29]). It takes place once a week for three hours in 10 sessions over a period of 10–12 weeks. Key quality features are multiprofessionalism, integrated cooperation, evidence-based, and a structured interdisciplinary approach using a defined therapy manual including standard materials (worksheets, presentation slides, exercise instructions, etc.; Fig. 1).

The A‑IMPT consists of a total of five modules—each lasting 2 weeks—which are grouped thematically (Fig. 1). Initially, the focus is on providing knowledge, which is expanded through the development of specific therapy goals and practical exercises and integrated into the patient’s world of experience. The overall focus is on the joy of movement and the discovery and protection of one’s own physical and psychosocial resources. Therapeutic interventions are introduced right at the beginning, including the development of strategies for coping with pain and everyday life, in order to support the transfer to everyday life. Each session has educational parts and ends with practical content (exercises, discussion, tasks to do at home), which is revisited in the following week and transferred to the new topic. The content delivered can be found in Table 2*.*

**Table 2:** Contents of education within the framework of the new A‑IMPT

Profession	Medicine	Physiotherapy		Psychology
*Week 1*	Biopsychosocial pain model, general treatment goals			
	Neurophysiology	–		–
*Week 2*	Anatomy, musculoskeletal system		–	
	–	Patient-specific functional scale, graded activity		Resilience (part I)
*Week 3*	Stress and stress management			
	Autonomous nervous system	–		–
*Week 4*	Stress and strain regulation, individual goals			
	–	–		Mindfulness
*Week 5*	–	Mobility and segmental stabilization		Cognition and pain, resilience (part II)
*Week 6*	–	Coordination, strength, endurance		Needs and emotions
*Week 7*	Health literacy, discussion on nutrition			
	–	Physical activity		–
*Week 8*	Sleep			
	Medication	Ergonomics		–
*Week 9*	Transfer-related goals, relapse prevention			
*Week 10*	Balance and transfer to everyday life			

The group sessions are supervised equally and simultaneously by psychological and physiotherapeutic colleagues; medical colleagues are also available for the group processes for approximately 10 h. In addition to an individual discussion with each profession, the patient receives a final consultation. Regular interdisciplinary team meetings are held every 2 weeks. Group and individual appointments, and the provision of materials are coordinated by the documentation assistants and/or nursing staff.

## Methodological procedures

Recommendations for reporting development processes for Theory of Change studies are summarized in Breuer et al. [6].

### Procedure for developing a Theory of Change for the A-IMPT

The development of a Theory of Change is method-neutral [10, 46]. For PAIN 2.0, literature research (scoping), expert surveys, and consensus-based processes in the project team were performed.

#### Step 1: starting point and objective of the new form of care: determination of the current and target status

According to current knowledge, people with recurring and ongoing pain are at risk of a chronification of their pain experience when risk factors are present (Table 1), resulting in physical and psychosocial impairments. Yet risk factors are not very helpful for the conceptualization of health care interventions; mechanisms underlying these risk factors that can be addressed through therapeutic interventions appear to be more relevant. At the same time, from the authors’ point of view, risk factors are more likely to be a consequence of inadequate health-related skills and actions. It can be assumed that certain skills and abilities such as health literacy (e.g., the ability to acquire relevant information on the development and maintenance of pain) and self-control skills (e.g., maintaining a regular daily routine despite recurring stressful experiences) play an important role, especially in the transition from recurring pain to a chronic form or worsening of ongoing pain. In particular, the functional maintenance of physical activity in the event of recurring pain requires, on the one hand, sufficient skills to obtain appropriate information and, on the other, the ability to adapt and review one’s behavior in terms of one’s own needs and well-being.

##### Focus: self-direction and self-regulation skills.

In psychological perspective, self-direction refers to the ability to make decisions, set one’s own goals and implement them despite challenges and internal or external resistance [28]. Self-direction encompasses awareness, will, and the ability to take into account personal needs and obstacles in order to achieve identifiable goals. Self-direction therefore refers to a certain type of knowledge that is based on conclusions drawn from given facts and rules and forms the prerequisite for ‘self-directed’ action [28]. Furthermore, it encompasses the capacity to cope with adversity and, most crucially, to consider individual needs, emotions, values, and interests when establishing objectives. Consequently, the overarching concept of self-direction is distinguished by two fundamental elements: the initial component, which encompasses the formation and sustenance of self-consistent objectives, represents *self-regulation*, while the subsequent component, which involves the pursuit of objectives guided by explicit intentions, pertains to processes of *self-control* [17]. The diverse maladaptive coping strategies observed in patients with chronic pain may, therefore, be explicable in terms of varying patterns of self-regulatory deficits [14] and improved through therapeutic interventions [38].

##### Focus: health literacy.

The European Health Literacy Consortium [42] defines the term “health literacy” as follows: “[…] encompasses the knowledge, motivation, and competence to access, understand, evaluate, and apply health information in order to make decisions in daily life regarding healthcare, disease prevention, and health promotion, and to maintain or improve quality of life.” This signifies that individuals with health literacy are capable of locating or acquiring health-related information, comprehending and assessing it, and utilizing it for themselves or their relatives. Furthermore, they possess the motivation, intention, and willingness to assume responsibility for their own health, assertiveness, the capacity to leverage social resources, and the ability to navigate the healthcare system. According to recent studies, over half of the German population report problematic or inadequate health literacy, and this has also deteriorated in recent years [39]. Individuals with a low level of education (78.3%), low social status (71.9%), advanced age (65.1%), and chronic pain (62.3%) are particularly affected. The data indicate that individuals with low health literacy tend to rate their health status more negatively, engage in fewer health-promoting behaviors, experience more frequent absences from work due to illness, have more regular contact with healthcare providers and utilize emergency medical services more frequently. Furthermore, the incorporation of health literacy into secondary prevention strategies aligns with broader health policy initiatives [39].

Concurrently, the prevalence of sedentary lifestyles is also on the rise [16]. Movement-related health literacy is a fundamental prerequisite for processing movement-related cognitive and sensorimotor information that is relevant to health, or for understanding and using health-related information during independent physical and sporting activities [44]. The model of movement-related health competence aims to establish the foundation for an “health-promoting physical activity” that extends well beyond the conventional focus on functional enhancement or restoration. The model places particular emphasis on relevant activities, aspects of participation, and person-related contextual factors. It posits three distinct domains: movement-related fundamental abilities and skills, control-related fundamental knowledge and abilities, and self-regulatory competence, which encompasses beneficial personal action characteristics and attitudes [8, 44]. It seems plausible to suggest that health competence is associated with the capacity to manage pain effectively, to prevent it from occurring, and to promote general wellbeing.

##### Focus: physical activity.

The World Health Organization [34] defines physical activity as movements of the body that are conducted through the expenditure of energy by the skeletal muscles. This encompasses all movements conducted during leisure, sporting activities, occupational pursuits, or domestic tasks. They may be undertaken of one’s own volition, offering pleasure, and/or fulfilling obligatory responsibilities. Physical activity has a beneficial effect on health at multiple levels. These include the total mortality rate, the prevention of cardiorenal metabolic disease, the enhancement of quality of life, and the prevention of depression [9]. Despite the positive effects on health, there has been a notable decline in the amount of time people spend engaging in physical activity [16]. In particular, among patient populations with chronic pain conditions, there is a notable reduction in the level of physical activity, which ought to be given specific attention as a crucial element of the therapeutic regimen [35]. Regular physical activity has numerous benefits for individuals with chronic pain. It has been shown to have a favorable impact on disease development and the underlying physiological processes. Additionally, it can help reduce the severity of symptoms, enhance physical functionality and resilience, improve psychological well-being, and elevate the quality of life in relation to one’s health [35].

##### The delineation of objectives for the A-IMPT.

A consensus was reached by representatives of the Deutsche Schmerzgesellschaft e. V. (online survey of the German Pain Society) on goals for an interdisciplinary intervention for individuals suffering from recurrent and ongoing pain who are also at risk of chronification [36]. These can be found in an adapted (condensed) form in Fig. 3. The attainment of these objectives is expected to contribute to an improvement in health literacy, self-regulation (in relation to pain and physical and psychological distress), and the maintenance or intensification of physical activity over the medium term. In the longer term, this should result in the prevention of pain chronification, including a reduction in existing pain intensity and pain-related functional impairment (primary outcomes, [29]) and in maintaining mental and social well-being, as well as the capacity for social participation (secondary outcomes, [29]).

#### Step 2: determine the active components for the stated objectives

##### Therapeutic effects induced by the A-IMPT.

**Fig. 2:**
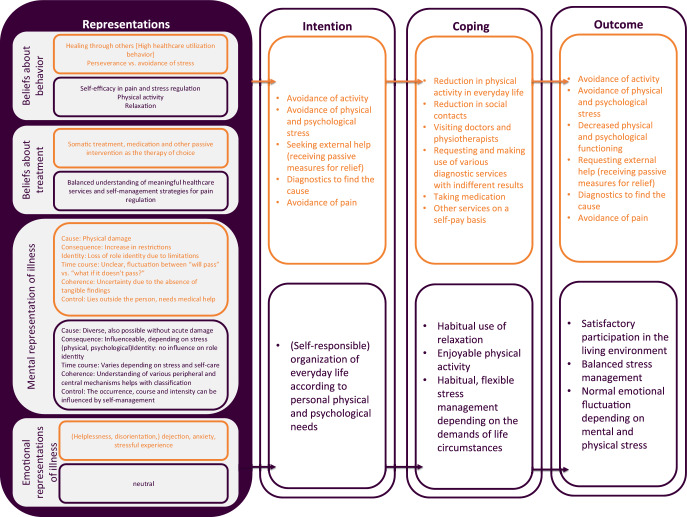
The Common Sense Model [22]. Adapted for the management of recurrent or persistent pain

At the level of systematic investigation, it appears that a combination of educational and physical activation programs may be a viable approach for the targeted audience [19, 43]. The subsequent conceptualization of the proposed model and the theory of change is informed by the Common Sense Model of Illness Representation, as extended by Hagger [22], which aligns with the aforementioned characteristics associated with pain chronification (Fig. 2). The currently available scientific findings regarding the risk factors presented in Table 1 are integrated into the theoretical considerations.

Individuals develop specific intentions for coping with illness based on their emotional and mental representations of the disease, including their beliefs about it. These result in coping strategies that, in turn, produce a specific outcome. In the case of patients experiencing recurrent and ongoing pain, the further management and the medium- and long-term development of associated physical, emotional, and social well-being are contingent upon the extent to which they possess sufficient elaborated and well-informed representations of their illness and their typical behavioral patterns. This level of competence directly builds on existing health literacy. As illustrated in Fig. 2, maladaptive characteristics (orange) are juxtaposed against potentially modifiable, more adaptive characteristics (lilac) that are targeted by the intervention.

The model was used to derive and identify and generate target group-specific information, self-monitoring tasks, and reflections within the context of therapy. The objective was to thus enhance general health literacy with regard to health behavior, the development of pain, and physical activity, as well as to initiate changes at the level of illness representations and beliefs.

It is assumed that the formation of intentions to adopt new behavioral patterns requires a change in illness representations, as well as the promotion of self-regulatory skills. The authors hypothesize that the bodily exercises under supervised self-monitoring and the material on regulating needs and emotions facilitate reflection and transformation with respect to self-regulation competence. The dissemination of exercises aimed at enhancing the vegetative balance or stabilizing the vegetative nervous system may also potentially contribute to an improvement in self-regulation functions.

Improving movement-related health literacy within the therapeutic context entails providing information, encouraging self-observation, offering concrete guidance on exercise routines, and facilitating the integration of these routines into daily life. By prioritizing enjoyment and pleasure in selecting physical activities and introducing low-threshold exercises, the motivation to maintain or enhance physical activity should be increased, leading to long-term improvements.

The comprehensive approach aims to achieve sustainable improvements in well-being, maintenance of participation and activity (secondary outcomes), and a reduction in pain-related function and pain experience (primary outcomes).

##### Contextual factors potentially influencing the effect of the new care model.

**Fig. 3:**
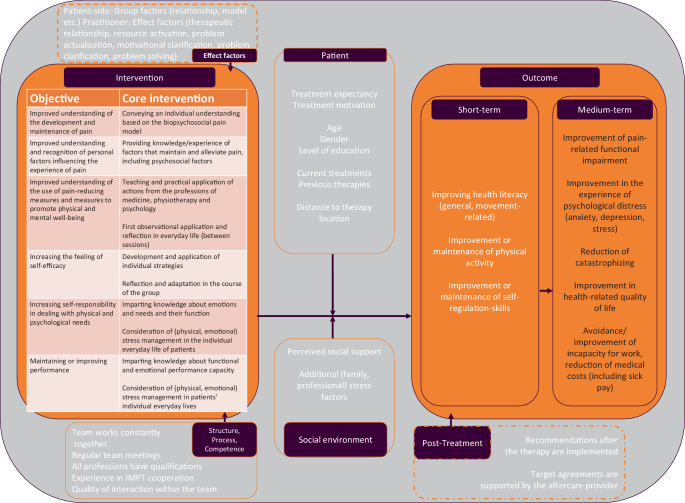
The new A‑IMPT care service in the (postulated) context of its execution (solid box lines: collected in the project, dashed box lines: not collected or indirectly collected in the project)

As illustrated in Fig. 3, a variety of contextual factors play a role in the execution of A‑IMPT. These factors can be classified into three categories: individual-related contextual factors, factors related to the patient’s environment, and factors related to the setting and the delivery of the new care model.

In consideration of the planned intervention, five areas of influence are identified (Fig. 3). As *patient-related context factors*, age, gender, and educational level [5], treatment expectancy and treatment motivation [11], the distance to the treatment location, previous therapies, and concurrent treatments outside of the new care model are of particular interest. *Context factors in the patient’s environment* relate to perceived support from social systems (e.g., partnerships, families) and additional psychosocial stressors [20]. *Context factors in the setting of the new form of care* pertain to the quality of interdisciplinary pain therapy provided by the team (e.g., stable collaboration, consistency in team composition, qualifications of team members). *Context factors over the course of the new care model* pertain to the quality of interaction during group therapy sessions, between patients and between patients and healthcare professionals (e.g., therapeutic factors such as therapeutic relationships, clarification of motivational, etc.) [21].

Finally, specific treatments post intervention and future communication primary care and specialist physicians may influence the extent to which the long-term goals agreed in the new care plan are implemented by patients. Unfortunately, this area of context factors cannot be assessed or influenced directly within the PAIN 2.0 project.

### Presentation of the effect model for outpatient interdisciplinary multimodal pain therapy

In accordance with the theoretical, evidence-based and therapeutic content considerations outlined above, the effect model for A‑IMPT is shown in Fig. 3.

### Research question and hypotheses

The evaluation concept for the effectiveness (efficacy) of the newly designed A‑IMPT care service is reported in detail elsewhere [29]. This is a clinically randomized study with a cross-over design. A net case number of *n* = 700 patients is planned, who will be monitored on 5 measurement points (quarterly between the start of therapy and 12 month later). Primary outcome parameters are pain intensity, pain-related disability and patient-related satisfaction [29].

In addition, the following hypotheses were formulated for the analysis and testing of the effect model:

- *Main hypothesis*: Participation in the A‑IMPT leads to a significant improvement in the *process variables* (physical activity, [movement-related] health literacy and flexible self-direction) in patients in the intervention group (compared to patients in the control group) from t_0_ to t_1_.
  1. A change in the process variables of the impact model takes place over the measurement times (t_0_ and t_1_).
  2. There is a difference in the expression of the process variables between the intervention and control group at t_1_.
- *Moderation hypothesis: Patient-related context factors *(age, gender, level of education, therapy expectancy, therapy motivation, distance to the PAIN 2.0-Center as well as previous therapies and concurrent treatments outside the new form of care), *context factors in the patient’s environment *(perceived support from the social system, additional psychosocial stress factors), *context factors in the setting of the new form of care* (quality of the implementation of interdisciplinary pain therapy by the team), and *context factors during the execution of the new form of care* have a moderating influence on the change in the process variables from t_1_ to t_2_.
- *Mediation hypothesis:* Higher values in the process variables at t_1_ causally explain the reduction in impairment (measured by the primary outcomes pain intensity and pain-related functional impairment) as a result of participation in the A‑IMPT.
- *Time hypothesis: *The improvement in the process variables will increase over the course of the intervention and remain stable in the long term, even after completion of the original 10-week therapy.

The online supplementary material “Constructs and Operationalization” contains a detailed description of the constructs, potential and selected survey instruments (including a detailed description) and corresponding test psychometrics for control variables and process variables.

### Operationalization and survey plan

**Fig. 4:**
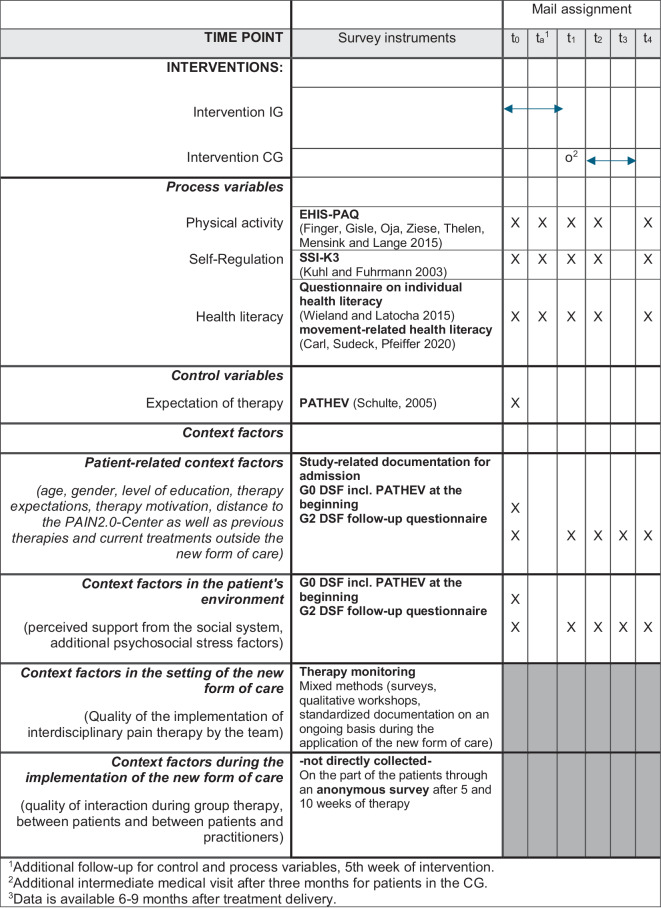
Process variables, survey instruments and measurement times (IG intervention group, CG control group, EHIS-PAQ European Health Interview Survey—Physical Activity Questionnaire, SSI-K3 Self-direction Inventory, BGK Questionnaire for assessing movement-related health literacy, DFS German Pain Questionnaire)

Figure 4 summarizes all postulated process and control variables including the planned survey instruments, their sources and survey dates.

In the course of the project, the process variables are recorded at five measurement points for both the intervention and the control group: start of therapy (t_0_), time of the 5th group appointment of the intervention group (t_a_), after 3 months (t_1_), after 6 months (t_2_) and after 12 months (t_4_) (Fig. 4). The control variables are collected at the start of therapy (t_0_), as are the personal environment factors; the context factors relating to the setting and implementation of the new form of care are collected with the help of comprehensive process monitoring (see Berger et al., 10.1007/s00482-024-00853-7).

### Analysis

The planned analysis of the PAIN 2.0 effect model takes into account the complexity of the intervention and comprises several analytical approaches [45].

The evaluation procedure for testing the effect model is based on a combination of quantitative methods. Within a multistep procedure, both descriptive statistics to characterize the sample and, depending on the requirements of the data, advanced statistical methods to investigate correlations and mediation [27] and moderation effects are used. Both single-factor and multifactorial analyses are performed in order to capture the complex relationships between the variables under investigation in accordance with the requirements of a complex intervention [47].

## Summary and outlook

Due to the complexity of the components of the new form of outpatient interdisciplinary multimodal pain therapy (including interdisciplinary group design, different professions with multimodal approaches, group-related variables) and the corresponding context (including the healthcare landscape in general, regional characteristics, patient-related factors, and the personal environment of the participants), consideration of the intended effect in the evaluation is relevant. The effect model for the A‑IMPT takes these factors into account and thus enables specific hypotheses for investigating the mode of action (in addition to their effectiveness in the context of the evaluation in the form of a clinical randomized study [29]). The explicit consideration of person-related (person-internal, e.g., treatment expectations, age, gender and environment-related, e.g., social support) and facility-related (e.g., quality of interdisciplinary cooperation) context factors in the form of control variables also points to aspects of the subsequent application of A‑IMPT in standard care.

Knowledge of these levels of contextual factors also makes it possible to make decisions regarding subgroup or control evaluations within the framework of the evaluation, which justify a better decision-making situation for the subsequent recommendation of the A‑IMPT from the perspective of the external evaluators. The causal hypotheses also provide indications of the extent to which the selected and designed interventions actually influenced the target parameters of health literacy and physical activity as well as the self-regulation skills of the participating patients. Concrete adjustments to the therapy manual are thus possible and evidence-based.

Challenges will arise on several levels in relation to the evaluation of the complex intervention of A‑IMPT. The number of different interventions, the interaction between practitioners and patients (previously unknown for interdisciplinary settings because it has not been investigated), but also between the practitioners and the patients themselves, the influence of the competencies of the persons involved (both practitioners and patients) and the verification of the implementation of the intervention adhering to the study protocol cannot be fully documented and analyzed in this abundance within the framework of the study. The effect model and the recording of operationalized parameters are only a small part of the reality of care. In principle, this study is at the beginning of an explicit evaluation of interdisciplinary care services for pain, which includes factors of team-based implementation more precisely in the study evaluation. Subsequent conclusions can therefore be understood as exploratory and, at best, as a first approximation to hypothesis and model building.

The novelty of the approach and the complex perspective on pain therapy in the context of PAIN 2.0 may require a primarily qualitative approach (e.g., theory-generating through grounded theory). However, with the focus of the funding framework of the Innovation Fund (primarily proof of effectiveness or efficiency), there are limited resources available for comprehensive qualitative surveys (and in particular their evaluation). For this reason, existing concepts, survey concepts, and selected questions were used in PAIN 2.0, as well as qualitative procedures in the sense of expert focus groups (in the form of workshops). However, this should not obscure the fact that much of what works and happens in this intervention is still unidentified (e.g., role of body awareness, motivation, quality of interaction in the team). This means that methods such as those required for complex and new interventions [4, 33] can only be implemented to a limited extent within the framework of this form of support due to time and financial constraints.

Furthermore, there are no theories and models known to us as a basis for the conception of effective models of interdisciplinary pain care. To the best of our knowledge, the complexity of group relationships between patients and practitioners and their dynamics in joint interaction have not yet been considered. PAIN 2.0 can make an initial contribution to the formulation of research questions and initial indications of relevant connections and thus contribute to later theory development—even though we address the deficit and thus point out the need for usable theories.

## Conclusion

A sustainable approach to newly designed interventions in the form of adaptations appears desirable in view of the long duration, high costs, and personnel costs for the development of complex interventions. The danger of a “pure” focus on effectiveness, which assesses interventions solely on the foundation of effect sizes and levels of significance as effective or ineffective, is that elaborately designed interventions are dropped. In the case of unconfirmed effectiveness, a consideration of the effects include the possibility of making sensible adjustments based on the work already carried out, which may increase effectiveness.

## Supplementary Information

Process and control variables

## Data Availability

Data collected as part of PAIN 2.0 are not publicly accessible due to the measures chosen to comply with data protection and can only be requested from the corresponding author upon justified request. After data entry, the data are stored in a data repository with controlled access at the German Pain Society. Data collection and entry are currently underway and will be completed at the end of the project.

## Declarations

For this article no studies with human participants or animals were performed by any of the authors. All studies mentioned were in accordance with the ethical standards indicated in each case.

The supplement containing this article is not sponsored by industry.
