# Supplementary material for: How does an intervention work?—English Version: Development of an effect model for a complex intervention to prevent recurring or persistent pain using the example of PAIN 2.0
Source: Schmerz. 2025 Jan 21;39(Suppl 2):77–88. doi: 10.1007/s00482-024-00860-8 (PMC12701007; doi:10.1007/s00482-024-00860-8)
Supplement: Supplementary file 1 — Process and control variables [file 482_2024_860_MOESM1_ESM.pdf]

Self-Direction and self-regulation skills	
**Construct (general)**	Self-direction skills
**Short description**	In everyday life, self-direction means acting consciously on one's own responsibility and on one's own initiative. From a psychological perspective, self-direction refers to the ability to make decisions, set one's own goals and implement them despite challenges and internal or external resistance (Kuhl, 1983, 1998, 2001). Self-direction includes awareness, will and the ability to take personal needs and obstacles into account in order to achieve identifiable goals. Self-direction therefore represents a certain type of knowledge that arises from conclusions drawn from given facts and rules and forms the prerequisite for 'self-determined' action (Kuhl, 2001, p. 133).<br><br>It also includes the ability to deal with difficulties and remain true to one's goals, as well as to take personal needs, feelings, values and interests into account when setting goals. This enables identification with the goal. Accordingly, the general concept of self-direction is divided into two components: The first component, i.e. the formation and maintenance of self-congruent goals, represents the so-called **self-regulation**. The second component - goal pursuit mediated by explicit intentions - refers to processes of **self-control** (Fröhlich & Kuhl, 2003, p. 222). The effective application of self-control depends on various conditions and circumstances.<br><br>People strive to maintain their psychobiological balance by setting goals that meet their needs. Chronic pain or physical limitations can prevent people from achieving their goals in the usual way (Karoly & Crombez, 2018). In response, some people begin an adaptation process to find new ways to meet their needs. Adapting goals or methods for achieving goals to one's own functional level, taking physical and emotional needs into account, can often lead to improved pain acceptance and a higher quality of life (Esteve et al., 2018; Ramírez-Maestre et al., 2019). In addition to pain acceptance, quality of life is also often recorded as an important outcome variable in interdisciplinary multimodal pain therapy (IMPT) (Kaiser et al., 2018). For an adaptation process to be successful, people must have the necessary self-direction skills to be able to achieve long-term and challenging goals. In his personality-system interaction theory (PSI theory; Kuhl, 2001), Kuhl describes psychological functions that enable people to set new goals (self-regulation) based on comprehensive self-representations and emotional needs and to pursue them despite internal and external resistance (self-control; Kuhl, 1983; 1998; 2001). Patients who can successfully manage their pain are likely to have the ability to switch appropriately between self-regulatory and self-controlling competencies (Van Damme & Kindermans, 2015; Karoly & Crombez, 2018).

Based on the comprehensive integration of basic scientific results from the field of volition, motivation and personality research, Kuhl models four macrosystems in his PSI theory that are involved in action control. There is a reciprocal exchange of information between the systems.

The activation of the four macrosystems is influenced by positive and negative affective states, with the relative ratio of the activation strength determining which system is significantly involved in the modulation of behavior and experience. Kuhl attributes the functions of self-control and self-regulation to the complex "intention and extension memory" systems located in the prefrontal cortex, which will be explained in more detail below (Kuhl, 2001).

Functional characteristics of self-regulation

Goals are created to meet one's own or others' needs. A system that represents such states in a comprehensive sense is required to guide the creation of goals based on one's own needs, emotional preferences and values. The generation of a general goal taking into account a variety of needs and feelings requires a parallel, holistic form of processing. Neuroscientific studies indicate that right-hemispheric systems tend to support holistic-implicit representations (Bradshaw, 1989). By comparing an achieved action result with the relevant part of the extension memory, even results that do not exactly match those consciously expected can be viewed as acceptable successes of self-control. This can lead to an increase in flexibility and positive self-efficacy expectations. The system must be able to override higher-level instructions, especially in threatening situations. In acute stress situations, the extension memory and thus access to the self is inhibited. The object recognition system is crucial here, as it is specialized in recognizing inconsistencies and giving priority to quick reactions and concrete goals when necessary. According to Kuhl, the development and maintenance of new intentions is based on partially unconscious processing of parallel neural networks that underlie self-representations. These highly inferential processing mechanisms serve to select action intentions that align with one's own motives, needs, and life experiences (the "self system"). In order to develop goals that are consistent with the self, people must be able to activate the psychological system that supports self-determination: extension memory. Access to extension memory is only granted when one is able to regulate negative affect and relax. According to Kuhl, negative affect, which often occurs in chronic pain patients, will activate the object recognition system. This system highlights mood-congruent perceptual aspects and isolates them from the overall context. In chronic pain patients, this mechanism leads to attention being strongly focused on pain-relevant stimuli and may cause pain sensations to be perceived more strongly than other sensations. Underdevelopment or limited access to the self-representational system under stress can lead to behavioral rigidity and compulsive behavior. Excessive inhibition

of the self-system can lead to the loss of all functions required for the emotional support of a self-selected goal (such as self-soothing, self-motivation, attentive search for further options for action or creative ways to redesign situations). Deficits in self-soothing can prevent the integration of painful experiences into existing networks of implicit self-representations (assimilation) or impair the modification of existing experience networks (accommodation).

Functional characteristics of self-control

Self-control refers to a system configuration in which explicit intentions stimulate actions and the influence of comprehensive self and context representations is weakened. According to Kuhl, support for goal realization is mainly realized through the interaction of intention memory and intuitive behavior control. Intentions must be maintained in a future-oriented subsystem of working memory until a suitable opportunity for realization is found and the required behavioral routines are sufficiently activated. Pre-activated motor programs must be counteracted to prevent premature execution until the parameters of adaptive and voluntary behavioral routines are specified and the appropriate opportunity for execution is given. An action-related representation only becomes an intention when it acquires the ability to suppress action-inhibiting processes (such as positive affect). If positive affect cannot be inhibited or this inhibition is not tolerated, there is a risk of acting impulsively. In order to take action, the inhibition must be removed again (initiative). When the load is high, i.e. when the intention memory is overloaded with many intentions, there is a risk of volitional inhibition (procrastination). The ability to activate the self-system is crucial for the inhibiting energy associated with the formation of intentions to be removed (self-motivation based on the feeling that what is desired matches one's own interests). Certain steps of actions must be put into a meaningful order, which requires the ability to plan. Consistently pursuing goals also requires the ability to concentrate in order to suppress irrelevant impulses.

Adaptive pain coping and self-direction skills

People with inadequate emotion regulatios skills have difficulties in self-regulation when they experience stress or threats. This can lead to them often not being able to find appropriate coping strategies for chronic pain. Depending on individual reaction predispositions to stress (the so-called "first reactions" or personal styles, which can be measured with the PSSI; Kuhl & Kazén, 1997), various self-regulation functions (the "second reaction") must be particularly well developed to enable adaptive coping with pain. For example, a patient with an easily excitable sensory reaction (an easy activation of the punishment system) must have well-developed self-soothing skills in order not to fall into catastrophizing thinking.

	A patient who is easily activated motorically (i.e. is impulsive and has an easily activated reward system) must have well-developed self-control skills in order not to constantly fall into dysfunctional, automated behavior patterns (such as excessive perseverance).<br><br>Different maladaptive coping strategies in patients with chronic pain could possibly be explained by different patterns of self-regulation deficits (Van Damme & Kindermanns, 2015; Esteve et al., 2016). Specific self-regulation skills can be specifically improved through certain physical or cognitive-behavioral therapeutic measures (Ritz-Schulte et al., 2008). An analysis of self-regulation skills could therefore help in the future to individualize the sequence of specific interventions in IMPT and thus increase the long-term effectiveness of therapy programs.<br><br>The authors assume that the body-related therapies and the content that addresses need/emotion regulation, as well as the teaching of exercises to improve the autonomic balance or stabilization of the autonomic nervous system, even lead to an improvement in self-regulation functions. An improvement in self-regulation should have an impact on various outcomes (catastrophization, quality of life, etc.). Based on the PSI theory, the authors see the self-control functions as an important mediating variable.
**Chosen Test**	Self-direction Inventory - SSI-K3
**Authors**	Kuhl, J., & Fuhrmann, A. (1998).
**Rationale for the selection:**	It may be possible to identify different maladaptive coping strategies in patients with chronic pain based on different patterns of self-direction deficits (Van Damme & Kindermanns, 2015; Esteve et al., 2016). Certain self-direction skills can be specifically improved by specific body-related or cognitive-behavioral therapeutic measures (Ritz-Schulte et al., 2008). An analysis of self-direction skills could potentially help to individualize the sequence of certain interventions in IMPT and thus optimize the long-term effectiveness of therapy programs.
**Description**	The Self-direction Inventory (SSI) and the short form used here (SSI-K3) are used to assess components of self-regulation and self-control and were developed in 1998 by Kuhl and Fuhrmann. Their reliability and validity have been well-tested (cf. Fröhlich & Kuhl, 2003). The instrument comprises 52 items; three subscales (4-level Likert scale) with 4 questions each: self-regulation (self-determination, self-motivation, self-calming), self-control (ability to plan, fear-free goal orientation, initiative, implementing intentions, concentration, action orientation) and self-

	access (coping with failure, self-awareness, integration, action orientation after failure). In addition, the extent of the current stress and threat is assessed, since the self-direction functions must always be interpreted in relation to the perceived stress. The greater the feeling of stress, the better certain skills must be developed.<br><br>Self-direction has already been shown to be significant in many fields of application, such as clinical psychotherapy, educational settings, sports, occupational, and organizational psychology. The SSI enables the assessment of an individual's functional components, providing a foundation for targeted interventions. [https://edoc.hu-berlin.de/bitstream/handle/18452/16834/lezinsky.pdf?sequence=1]
**Scales (Items)**	52 items; three subscales (4-level Likert scale) with 4 questions each:<br>self-regulation (self-determination, self-motivation, self-calming), self-control (ability to plan, fear-free goal orientation, initiative, implementing intentions, concentration, action orientation) and self-access (coping with failure, self-awareness, integration, action orientation after failure).<br><br>In addition, the extent of the current stress and threat is assessed, since the self-direction functions must always be interpreted in relation to the perceived stress. The greater the feeling of stress, the better certain skills must be developed.
**Response format**	"not at all", "somewhat", "mostly", "extremely"
**Standard values**	There are currently no standard values for pain patients or the general population, as it is more suitable for measuring progression.
**Test psychometrics[1]**	
**Content Validity**	–
**Construct Validity**	–
**Convergent Validity**	The subscales of the SSI-K3 demonstrate structural similarity to questionnaires assessing emotion regulation due to the significance of affect-regulatory processes. Unpublished data from a sample of fibromyalgia patients (N=40) show that the SSI subscales "Self-regulation" and "Self-access under stress" correlate with the questionnaire for standardized self-assessment of emotional competencies (SEK-27, Berking & Znoj, 2011) with r = 0.7 to r = 0.8 (Ramasawmy et al., in preparation).

[1] Based on the COSMIN specifications, see below the overall table

**Criterion Validity**	Self-guidance is mapped with apparent validity. Validation studies conducted show a strong alignment between the questionnaire measures and objective methods for assessing the individual self-control functions. Correlations were found between hesitation or energy deficit (lack of energy/lack of motivation) and longer response times when switching actions under self-control (Dibbelt, 1997), or between external control and superior performance in externally chosen activities compared to self-selected activities (Kuhl & Kazén, 1994). These data refer to the extended version of the SSI, which consists of 190 items, assigned to 32 functional components and six additional scales, yielding 5 items per scale.
**Reliability – Interrater**	The TOP-Diagnostics manual by IMPART GmbH contains indications of internal consistency (Cronbach's alpha = 0.72 - 0.82) and test-retest reliability (r = 0.51 - 0.81) (J. Kuhl & Henseler, 2004; J. Kuhl & Kaschel, 2004).
**Change sensitivity**	The instrument has been used in several studies in the rehabilitation context to assess the effectiveness of interventions, showing varying effect sizes (ranging from weak to strong effects), suggesting sensitivity to change. For example, studies by Bronowski (2010) and Schmädeke et al. (2019) demonstrate this.
**Test criticism**	A major methodological challenge concerns the valid assessment of "self-access under stress" and implicit emotion regulation, which play a crucial role in the PSI theory. The PSI theory defines the "self" as a comprehensive network that operates based on connectionist principles and parallel processing. The "results" of these extensive processing activities become conscious as a holistic "gut feeling" or intuition. The specific experiences that led to this intelligent gut feeling are largely not accessible to consciousness. People with limited self-access, due to constant tension or an inability to adequately downregulate negative affect, are often unaware of their own self-alienation. It is evident that implicit self-regulatory processes cannot be captured using introspective methods (but rather through psychophysiological methods such as heart rate variability). Therefore, the SSI-K3 likely only captures consciously representable functions of the "self."
**Further tests in the shortlist that were excluded:**	

Scale of General Self-Efficacy (SWE)	
**Authors**	Schwarzer, R. und Jerusalem, M. (1999)
**Description**	A self-assessment tool with 10 items designed to measure general optimistic self-beliefs. It assesses the optimistic expectation of competence, meaning the confidence in one's ability to cope with difficult situations, attributing success to one's own competence. Specific variants have been published since, such as the school-related SWE (Jerusalem & Satow, 1999; Ralf Schwarzer, Jerusalem & Mittag, 1999; Satow, 1999), the teacher-SWE (Schmitz & Schwarzer, 2000), and the collective teacher-SWE (Schwarzer & Jerusalem, 1999; Schwarzer & Schmitz, 1999).<br><br>The instrument is based on the concept of self-efficacy (perceived self-efficacy) introduced by Bandura in 1977, which forms a key aspect of his social-cognitive theory (Bandura, 1997). It was originally conceived as a situation-specific construct, though the degree of specificity can vary. General SWE represents an extreme end and is based on the assumption that people can attribute their success and failure experiences to themselves and generalize from them. This involves not only inductive processes (from specific to general) but also deductive processes (from general to specific). The scale measures the subjective belief in the ability to successfully cope with challenging or demanding situations on one's own. It considers new or difficult situations from all areas of life, as well as barriers to overcome. SWE is intended to predict constructive life management (Jerusalem, 1990; Schwarzer, 1994).
**Psychometric properties**	When comparing 23 nations, the internal consistencies (Cronbach's alpha) ranged between .76 and .90, with German samples consistently falling between .80 and .90.<br><br>The validity is supported by numerous correlation findings with other relevant variables. Strong positive correlations were found with dispositional optimism and job satisfaction, and strong negative correlations with anxiety, depression, burnout, stress assessments (threat, loss), and so on. In studies with former East German emigrants, physical complaints and depression were predicted over a two-year period, and in patients with heart disease, recovery and postoperative quality of life were well predicted after six months.
**Exclusion reasons**	The SWE is considered to have low sensitivity to change and is therefore rather unsuitable for the purposes of the study.

TGP und FGA - Tenacious Goal Pursuit and Flexible Goal Adjustment	
**Authors**	**Brandstädter, J. und Renner, G. (1990).**
**Description**	The questionnaire is based on a model of coping performance by Brandstätter and Renner, which defines two distinct modalities for coping performance: the assimilative and the accommodative mode. The assimilative mode is understood as changing situations in accordance with personal goals, while the accommodative mode refers to adjusting personal wishes and goals to situational circumstances. Both scales are associated with high life satisfaction, low levels of depressive mood, and internal control beliefs. The questionnaire consists of 30 items.
**Psychometric properties**	*Objectivity*: Given in administration, instructions, and evaluation<br>*Reliability*: Internal consistency of both scales is satisfactory, Cronbach's alpha between .80 and .83<br>*Validity*: No information provided
**Exclusion reasons**	The questionnaire on coping with problems primarily focuses on the concept of coping strategies. The SSI-K3 captures a broader range of aspects of self-regulation and has thus been preferred.

Citations

<u>Literature on the construct of self-direction – Self-Direction Inventory (SSI-K3):</u>

Becker, S., Navratilova, E., Nees, F., Van Damme, S. (2018). Emotional and Motivational Pain Processing: Current State of Knowledge and Perspectives in Translational Research. Pain Research and Management. DOI: 10.1155/2018/5457870

Bradshaw, J.L., (1989). Hemispheric specialization and psychological function. Cichester, England: Wiley. (Buch)

Bronowski, I. (2010). Der Effekt der Alexander-Technik auf die Selbststeuerungskompetenzen und -effizienz. Diplomarbeit. Universität Koblenz-Landau, Campus Landau. Fachbereich Psychologie. (verfügbar unter: http://www.alexandertechnikschule.de/studien/ATundSelbststeuerung.pdf)

Dibbelt, S. (1997). Wechseln und Beibehalten von Zielen als Subfunktionen der Handlungskontrolle [Change and maintenance of goals as functional components of action control]. Dissertation. University of Osnabrück, Germany. https://doi.org/10.1037/0096-3445.136.4.593

Esteve, R., López-Martínez, A.E., Peters, M.L., Serrano-Ibáňez, E.R. et al. (2018). Optimism, Positive and Negative Affect, and Goal Adjustment Strategies: Their Relationship to Activity Patterns in Patients with Chronic Musculoskeletal Pain. Pain Research and Management. DOI: 10.1155/2018/6291719

Fröhlich, S. M. & Kuhl, J. (2003). Das Selbststeuerungsinventar: Dekomponierung volitionaler Funktionen: In F. Rheinberg & J. von Stiensmeier-Pelster (Hrsg.), Diagnostik von Motivation und Selbstkonzept. Jahrbuch der pädagogisch-psychologischen Diagnostik. Tests und Trends. Neue Folgen, Bd. 2 (S. 221-257). Göttingen: Hogrefe.

Karoly, P & Crombez, G. (2018). Motivational Perspectives on Chronic Pain: Theory, Research, and Practice. New York: Oxford University Press.

Kästner, A. und Petzke, F. (2024). Personality systems interactions theory: an integrative framework complementing the study of the motivational and volitional dynamics underlying adjustment to chronic pain. *Front. Pain Res.* 5:1288758.

Kuhl, J. (1983). Motivation, Konflikt und Handlungskontrolle. Berlin: Springer. (Buch)

Kuhl, J., & Fuhrmann, A. (1998). Decomposing self-regulation and self-control: The Volitional Components Inventory. In J. Heckhausen & C. S. Dweck (Eds.), Motivation and self-regulation across the life span (p. 15–49). Cambridge University Press.

Kuhl, J., & Kazén, M. (1994). Self-discrimination and memory: State orientation and false self-ascription of assigned activities. Journal of Personality and Social Psychology, 66(6),

Kuhl, J. (2001). Motivation und Persönlichkeit. Göttingen: Hogrefe. (Buch)

Kuhl, J. (2005). TOP-Manual. Universität Osnabrück. Verfügbar unter www.impart.de

Lenzinsky, D. (2010). Förderung der Selbststeuerungskompetenz von übergewichtigen Jugendlichen zur Umstellung des Gesundheitsverhaltens Wirksamkeitsprüfung des Interventionsprogramms S.T.E.P.S. Responderanalysen und Prädiktoren des Interventionserfolgs. Dissertation, Institut für Sportwissenschaft Abteilung Sportpsychologie und Gesundheitswissenschaften der Humboldt-Universität zu Berlin: Berlin. (verfügbar unter: https://edoc.hu-berlin.de/bitstream/handle/18452/16834/lezinsky.pdf?sequence=1)

Ramirez-Maestre, C., Esteve, R., López-Martínez, A.E., Serrano-Ibáñez, E.R. et al. (2019). Goal Adjustment and Well-Being: The Role of Optimism in Patients with Chronic Pain. Annals of Behavioral Medicine, 53 (7),

Ritz-Schulte, G., Schmidt, P., Kuhl, J. (2008). Persönlichkeitsorientierte Psychotherapie.Göttingen: Hogrefe.

Van Damme, S. & Kindermans, H. (2015). A self-regulation Perspective on Avoidance and Persistence Behavior in Chronic Pain: New Theories, new Challenges? The Clinical Journal of Pain, 21 (2), 115-122.

Literature on Tenacious Goal Pursuit and Flexible Goal Adjustment:

Brandstädter, J. und Renner, G. (1990). Tenacious goal pursuit and flexible goal adjustment: explication and age-related analyses of assimilative and accommodative strategies of coping, Psychology of Aging, 5, 58-67.

Gärtner, A. (2021). Kognitive Leistungsfähigkeit bei Patienten mit chronischen Schmerzen. Eine Untersuchung im Rahmen einer interdisziplinären multimodalen schmerztagesklinischen Behandlung am UniversitätsSchmerzCentrum Dresden. Medizinische Fakultät Dresden.

Literature on the Scale of General Self-Efficacy (SWE):

Bandura, A. (1997). *Self-efficacy: The exercise of control.* New York: Freeman.

Jerusalem, M. (1990). *Persönliche Ressourcen, Vulnerabilität und Streßerleben.* Göttingen: Hogrefe.

Jerusalem, M. & Mittag, W. (1999). Selbstwirksamkeit, Bezugsnormorientierung, Leistung und Wohlbefinden in der Schule. In M. Jerusalem & R. Pekrun (Hrsg.), *Emotion, Motivation und Leistung* (S. 223-245). Göttingen: Hogrefe.

Jerusalem, M. & Satow, L. (1999). Schulbezogene Selbstwirksamkeitserwartung. In R. Schwarzer & M. Jerusalem, (Hrsg.), *Skalen zur Erfassung von Lehrer- und Schülermerkmalen* (S. 15). Berlin: Freie Universität Berlin.

Satow, L. (1999). Zur Bedeutung des Unterrichtsklimas für die Entwicklung schulbezogener Selbstwirksamkeitserwartungen. Eine Mehrebenenanalyse mit latenten Variablen. *Zeitschrift für Entwicklungspsychologie und Pädagogische Psychologie, 31* (4), 171-179.

Schmitz, G. S. & Schwarzer, R. (2000). Selbstwirksamkeitserwartung von Lehrern: Längsschnittbefunde mit einem neuen Instrument. *Zeitschrift für Pädagogische Psychologie*, *14* (1), 12-25.

Schwarzer, R. (1994). Optimistische Kompetenzerwartung: Zur Erfassung einer personalen Bewältigungsressource. *Diagnostica, 40* (2), 105-123.

Schwarzer, R. & Jerusalem, M. (1999). Skalen zur Erfassung von Lehrer- und Schülermerkmalen. Dokumentation der psychometrischen Verfahren im Rahmen der Wissenschaftlichen Begleitung des Modellversuchs Selbstwirksame Schulen. Berlin: Freie Universität Berlin.

Schwarzer, R. & Schmitz, G. S. (1999). Kollektive Selbstwirksamkeitserwartung von Lehrern. Eine Längsschnittstudie in zehn Bundesländern. *Zeitschrift für Sozialpsychologie, 30* (4), 262-274.

Construct (general)	Physical Activity
**Short description**	The World Health Organization [WHO, 2018] defines physical activity (PA) as body movements performed by skeletal muscles that expend energy. This includes all forms of movement carried out during leisure time, sports, work, or household tasks. These movements can be undertaken voluntarily and enjoyed or as part of essential daily living activities.<br>Physical activity (PA) has numerous positive effects on health across various dimensions, such as reducing overall mortality, preventing cardiometabolic diseases, improving quality of life, and decreasing the prevalence of depression [PAGAC, 2018; Fiuza-Luces et al., 2013]. The compelling evidence supporting the health benefits of PA aligns closely with the contemporary understanding of health, which is not limited solely to physical-functional aspects but extends to biopsychosocial dimensions. These dimensions also focus on socio-ecological conditions beyond the individual level.<br>Despite the overwhelming arguments in favor of physical activity and its extraordinary health benefits, people are becoming increasingly sedentary. Froböse et al. [2018] reported that physical inactivity is on the rise. While 60% of respondents in 2010 met the minimum recommendations for health-enhancing PA, only 43% achieved this benchmark in 2018. These recommendations are based on the guidelines established by the WHO [2010]. According to these guidelines, adults should engage in at least 150 minutes of moderate-intensity aerobic PA per week (e.g., brisk walking; 3-6 METs, 50-60% of individual capacity), 75 minutes of vigorous-intensity aerobic PA per week (e.g., jogging; >6 METs, 70-80% of individual capacity), or any combination of these two options. Additionally, muscle-strengthening activities should be performed on at least two days per week [Rütten and Pfeifer, 2016].<br>The central message of the WHO [2020] is that even small increases in regular PA have health-promoting effects. This point is particularly important for individuals who do not currently meet the WHO recommendations and may feel demotivated by what they perceive as unattainable requirements.<br>To assess improvements in PA over time, evaluate the impact of interventions designed to promote PA, or determine the health benefits of PA, it is essential to use appropriate questionnaires that measure PA.<br>Numerous questionnaires have been developed to measure PA, with varying levels of psychometric quality. Some have been specifically designed for particular subgroups or settings, while others were created because researchers were either unaware of existing instruments or dissatisfied with those available. Van Poppel and colleagues conducted a literature review in 2010 using MEDLINE, EMBASE, and SportDiscus to evaluate and compare questionnaires assessing

physical activity (PA) in adults. The selection of articles, data extraction, and quality assessment were performed by two independent reviewers. The quality and results of the studies were evaluated using the Checklist for the Appraisal of Physical Activity Questionnaires (QAPAQ). Among the 85 questionnaires included, the psychometric properties were generally poor, with most lacking information on content validity, and construct validity and reliability were found to be relatively low. The authors concluded that there is a lack of standardization in PA questionnaires, and none of the identified questionnaires or types of questionnaires were superior for assessing PA. Therefore, they could not recommend any specific questionnaire unequivocally. They suggested using a questionnaire aligned with the current PA guidelines that measures duration and frequency and assesses PA in different domains (work, home, transportation, leisure, and sports) [van Poppel et al., 2010].

Sember et al. (2020) investigated, in their systematic review and meta-analysis, the test-retest reliability, concurrent validity, and criterion validity of the most commonly used international PA questionnaires: the International Physical Activity Questionnaire (IPAQ-SF), the Global Physical Activity Questionnaire (GPAQ), and the European Health Interview Survey-Physical Activity Questionnaire (EHIS-PAQ). The quality of the 20 included studies was assessed using the QAPAQ checklist. The meta-analysis found that moderate to vigorous physical activity (MVPA) was the most relevant outcome for assessing PA levels, as no publication bias was detected for any measurement properties. Test-retest reliability was moderately high (rw = 0.74), criterion validity was moderate (rw = 0.41), and concurrent validity was moderately high (rw = 0.72). In summary, in cases where self-reported PA assessment is necessary, the selected questionnaires are recommended for use with the adult population in the EU.

Particularly for patients with (chronic) pain, PA levels are reduced and should receive special focus as part of therapy [Pedersen and Saltin, 2015; Swedish National Institute of Public Health and Professionals Associations for Physical Activity, 2010; Rütten and Pfeiffer, 2016; Geneen et al., 2017]. The numerous health benefits of PA for individuals with chronic pain include favorable effects on pathogenesis and pathophysiology, symptom alleviation, improved physical function and resilience, enhanced psychosocial well-being, and better health-related quality of life [Pedersen and Saltin, 2015; Swedish National Institute of Public Health and Professionals Associations for Physical Activity, 2010]. Geneen et al. (2017) emphasized that PA programs need to be evaluated to identify factors determining their success or failure. In particular, extended follow-up periods are critical and should be taken into account [ibid.].

In the VAPAIN project (Validation and Application of a patient-relevant core outcome set to assess the effectiveness of multimodal pain therapy), PA was identified as a relevant therapeutic goal in interdisciplinary multimodal pain therapy (IMPT) [Kaiser et al., 2018]. The aim of the study was to develop a core outcome set (COS) with patient-relevant outcome domains for chronic pain in clinical IMPT studies. The COS recommendations were based on a Delphi study

	that included multiple online surveys and an in-person meeting involving all relevant stakeholders, including patient representatives. Among the domains of the COS is Physical Activity (PA).
**Chosen Test**	**EHIS-PAQ** (European Health Interview Survey – Physical Activity Questionnaire)
**Author**	**[Robert-Koch-Institut, 2017]**
**Rationale for the selection:**	The EHIS-PAQ is a domain-specific questionnaire for assessing physical activity (PA) that is shorter than the GPAQ[2] and IPAQ and was specifically developed for health surveys. The EHIS-PAQ has been tested in various regions and cultural contexts across Europe. While the IPAQ evaluates overall PA levels on a MET (metabolic equivalent) basis, summing all moderate and vigorous activities performed during a day, the EHIS-PAQ focuses on PA in health-relevant settings (work, transportation, leisure). From the perspective of European health monitoring, a setting-specific approach is preferred because the health benefits of PA vary across different domains [Savinainen et al., 2004; Schibye et al., 2001], health promotion measures and interventions aimed at improving PA often adopt domain-specific strategies [Kahlmeier et al., 2014; WHO, 2013; WHO, 2014], and it is easier for respondents to recall activities in specific settings than their total PA (as fewer activities need to be remembered and summed) [Ainsworth et al., 2012]. This reduces the mental effort (calculation and memorization) required from respondents, making activities easier to recall and potentially reducing recall bias [Ainsworth et al., 2012]. Compared to the IPAQ-SF, the EHIS-PAQ shows higher reliability and convergent validity, while criterion validity is similar. However, the sensitivity to change has not yet been investigated for either questionnaire.
**Description**	The EHIS-PAQ is the revised version of the IPAQ-SF and is based on questions that have already been used in large-scale health surveys. It also shares similarities with the GPAC. The EHIS-PAQ was developed with respondents in mind. It covers three domains of physical activity: work-related, transportation-related, and leisure-related physical activity. The EHIS-PAQ distinguishes between "aerobic" and "muscle-strengthening" physical activity, enabling an estimation of adherence to health-promoting physical activity recommendations.
**Scales (Items)**	8 items (work/leisure) + cycling for transportation per week + number of days per week engaging in muscle-strengthening activities

[2] The GPAQ (Global Physical Activity Questionnaire) assesses physical activity across all domains of life (work, transportation, and leisure) using 16 questions. The GPAQ serves as an intermediary instrument between the short (IPAQ-S) and long (IPAQ-I) versions of the IPAQ, avoiding interpretation discrepancies that may arise when using the IPAQ questionnaire [Bull et al., 2009].

**Response format**	**The response format varies depending on the question.**
**Standard values**	/
**Test psychometrics**[3]	
Content Validity	not investigated
Construct Validity – Convergent Validity	IPAQ-LF (work, transportation, leisure) – EHIS (work, transportation, MVPA, HEPA[4]) p=0,45-0,64 [Baumeister et al., 2016]
Criterion Validity	With accelerometry (MVPA, HEPA) p=0.35-0.43 [Baumeister et al., 2016]
Reliability – Interrater	ICC[5]=0,55 (range: 0,43-0,73) [Baumeister et al., 2016]
**Change sensitivity**	not investigated
**Test criticism**	Limitation of the setting-specific approach: For some individuals, it may be challenging to define the settings (e.g., leisure time for retirees).
**Further tests in the shortlist that were excluded:**	
**BSA-F** *(Movement and Physical Activity Questionnaire)*	
**Author**	**[Fuchs et al., 2015]**
**Description**	The BSA questionnaire is an instrument for assessing physical activity and sports participation in adults. It is a self-administered questionnaire that captures physical activity in the dimensions of frequency, duration, intensity, and type, and it includes specific subcategories of physical activity, such as movement activity in work and leisure, as well as sports participation. The questionnaire has been used in several studies [including Fuchs et al., 2011; Fuchs et al., 2015; Gerber et al., 2010; Klaperski et al., 2013].
**Psychometric properties**	*Validity – Concurrent and Prognostic:* Cycloergometric parameters of endurance performance (e.g., performance at the Individual Anaerobic Threshold p [IAS] and calculated VO2max) were used as validation criteria, collected in the context

[3] Oriented according to the COSMIN guidelines, see below the overall table.
[4] MVPA: moderate to vigorous physical activity; HEPA: Health-Enhancing Physical Activity
[5] Intraclass Correlation Coefficient

	of a randomized controlled intervention study (N = 118). Results: The overall activity index significantly correlated with r = .32 and r = .34 with the performance parameters VO2max and p [IAS], respectively (concurrent validity); the sport activity index significantly ($p < .01$) predicted the endurance performance measured 12 weeks later (prognostic validity) [Fuchs et al., 2015].<br><br>*Change Sensitivity:* Not investigated<br><br>*Reliability:* Not investigated
**Exclusion reasons**	The focus of the BSA questionnaire is more on the type and intensity of physical activity rather than providing a comprehensive overview of activity levels.<br>A potential issue with Block 1, which addresses occupational physical activities, could arise from non-working individuals being unable to provide an assessment. Additionally, Blocks 5 and 6 include questions about sports activities, although sports, according to the definition mentioned above, are not equivalent to physical activity. Therefore, the questionnaire was excluded.
**Freiburg Questionnaire on Physical Activity (Freiburger Fragebogen zur körperlichen Aktivität)**	
**Author**	[Frey et al., 1999]
**Short description**	The Freiburg Questionnaire collects information on everyday potentially health-promoting activities across different target groups using 12 questions. These include basic, leisure, and sports activities (e.g., physical strain at work; everyday activities such as walking/cycling or climbing stairs; sports activities or dancing and bowling). The questionnaire summarizes activities from the past week or month (self-assessment), which are then converted into hours per week during evaluation.<br>Additionally, questions are included on the duration of daily relaxation and sleep phases, self-assessment of one's activities compared to peers, and evaluation of physical condition. Responses are given on a 5- or 7-point scale. The questionnaire also collects personal data (gender, age, height, and weight). The results focus on total activity time as well as body weight-adjusted energy expenditure per week (total expenditure).
**Psychometric properties**	80 participants, aged between 18 and 78 years (50 men, 30 women)

	Validity:<br>- Correlation of maximal oxygen uptake with documented activity levels: Positive correlation with age (r=0.683, p<0.01), amount of sports activity (r=0.395, p<0.05), and an inverse correlation with the amount of basic activity (r=0.337, p>0.05).<br>- Due to the strong correlation between maximal oxygen uptake and age, partial correlation coefficients were calculated while controlling for age. Here, no relationship between maximal oxygen uptake and the amount of basic activity was found, while the correlation between maximal oxygen uptake and sports activity became more pronounced (r=0.422, p>0.01).<br>- Pearson correlation coefficients between "self-assessment of activity" and activity levels reported in the questionnaire: Participants who rated themselves as more active than their peers were indeed more active - sports activity: r=0.334, p<0.01; total activity: r=0.282, p<0.05.<br><br>*Reliability - Test-Retest:*<br>- Pearson correlation coefficients ranged between 0.998 and 0.751.<br>- Test-retest data variability was examined using reproducibility coefficients (2σ): e.g., basic activity (2.5 after 15 days, 10.1 after 6 months), leisure activity (1.2 after 14 days, 8.3 after 6 months), sports activity (1.7 after 14 days, 6.0 after 6 months).<br><br>*Change sensitivity:* Not investigated.
**Exclusion reasons**	The Freiburg Questionnaire focuses on the total activity time performed and overall energy expenditure. It includes activities that may not apply to everyone (e.g., leisure activities like dancing and bowling), which, if not performed, are counted as 0 minutes in the total activity time. Unlike the BSA, the Freiburg Questionnaire places less emphasis on the type and intensity of activities but still considers a very selective range of basic, leisure, and sports activities, as well as the total energy expenditure, which is not particularly relevant in the context of the above-described construct of physical activity.
**IPAQ-SF[6] german**	

[6] International Physical Activity Questionnaire-Short Form

**Author**	**[Craig et al., 2003]**
**Short description**	The IPAQ-SF German collects information on the number of days and duration of vigorous physical activity, moderate physical activity, and walking, as well as the duration of sitting on weekdays over the past seven days. It thus provides an overview of the overall level of physical activity. The questionnaire was used in the first EHIS wave. It is the short version of the IPAQ-LF.
**Psychometric properties**	*Reliability:*<br>*P = 0.22–0.54 [Rütten et al., 2003]*<br>*IPAQ-SF-S7T (for telephone interviews, response items slightly differ from the self-administered SF version)*<br>*R = 0.43–0.6 [Mäder et al., 2006] (for telephone interviews, response items slightly differ from the self-administered SF version)*<br><br>*Validity:*<br>*Not examined in German-speaking populations*<br><br>*Change Sensitivity:*<br>*Not investigated*<br><br>*IPAQ-S (original questionnaire, English):*<br>*Reliability:*<br>*ICC = 0.3–0.97 [Poppel et al., 2010]*<br>*P = 0.25–0.99 (predominantly > 0.7) [Craig et al., 2003]*<br><br>*Convergent Validity:*<br>*P = 0.11–0.46 [Joseph et al., 2021; Norway, patients with osteoarthritis]*
**Exclusion reasons**	A retrospective evaluation of the use of the IPAQ-SF revealed that 60–70% of respondents (depending on the physical activity question) indicated that it should be "removed," "completely revised," or "adjusted" [Robert Koch Institute, 2011].

	Additionally, participants reported difficulties in distinguishing between different intensity levels of physical activity, estimating the duration of activities they typically perform more or less unconsciously, such as walking and sitting, and combining multiple activities to answer a single question about overall physical activity.<br>The IPAQ-SF is the predecessor of the EHIS-PAQ.

Citations

Literature on the construct of physical activity:

Physical Activity Guidelines Advisory Committee (PAGAC) (2018) Physical Activity Guidelines Advisory Committee Scientific Report. U.S. Department of Health and Human Services, Washington, DC

Fiuza-Luces C, Garatachea N, Berger NA et al (2013) Exercise is the real polypill. Physiology (Bethesda), 28(5): 330-358

Froböse I, Biallas B, Walmann-Sperlich B (2018) Der DKV-Report 2018 „Wie gesund lebt Deutschland?“. Deutsche Krankenversicherung, Düsseldorf.

WHO (2010) Global recommendations on physical activity and health. WHO, Geneva

Rütten A, Pfeifer K (2016) Nationale Empfehlungen für Bewegung und Bewegungsförderung. Bundeszentrale für gesundheitliche Aufklärung, Köln

World Health Organization (WHO) (2018) Global action plan on physical activity 2018–2030: more active people for a healthier world. https://apps.who.int/iris/rest/bitstreams/1138597/retrieve. Zugegriffen am 29.10.2021

World Health Organization (WHO) (2020) WHO guidelines on physical activity and sedentary behaviour. World Health Organization, Geneva

van Poppel MN, Chinapaw MJ, Mokkink LB et al (2010) Physical activity questionnaires for adults: a systematic review of measurement properties. Sports Med. 40(7):565–600

Sember V, Meh K, Soric M et al (2020) Validity and Reliability of International Physical Activity Questionnaires for Adults across EU Countries: Systematic Review and Meta Analysis. In: International Journal of Environmental Research and Public Health 17, 7161

Swedish National Institute of Public Health and Professionals Associations for Physical Activity (2010): Physical activity in the prevention and treatment of disease. Swedish National Institute of Public Health, Östersund

Pedersen BK, Saltin B (2015) Exercise as medicine – evidence for prescribing exercise as therapy in 26 different chronic diseases. In: Scandinavian

Journal of Medicine & Science in Sports 25 (Suppl 3):1–72

Geneen LJ, Moore AR, Clarke C et al (2017) Physical activity and exercise for chronic pain in adults: an overview of Cochrane Reviews. In: Cochrane Database Syst Rev. 24, 4 (4)

Kaiser, U., Kopkow, C., Deckert, S. et al (2018) Developing a core outcome domain set to assessing effectiveness of interdisciplinary multimodal pain therapy: the VAPAIN consensus statement on core outcome-domains. *PAIN*, *159*(4), 673-683

Literature on available questionnaires:

Fuchs R, Klaperski S, Gerber M et al (2015) Messung der Bewegungs- und Sportaktivität mit dem BSA-Fragebogen: Eine methodische Zwischenbilanz. Zeitschrift für Gesundheitspsychologie 23:60-76

Fuchs R, Göhner W, Seelig H (2011) Long-term effects of a psychological group intervention on physical exercise and health: The MoVo concept. Journal of Physical Activity and Health 8:794–803

Gerber M, Fuchs R, Pühse U (2010) Einfluss eines Kurz-Interventionsprogramms auf das Bewegungsverhalten und seine psychologischen Voraussetzungen bei Übergewichtigen und Adipösen. Zeitschrift für Gesundheitspsychologie 18:159–169

Klaperski S, von Dawans B, Heinrichs M et al (2013) Does the level of physical exercise affect physiological and psychological responses to psychosocial stress in women? Psychology of Sport and Exercise 14:266–274

- - - - - - - - -

Frey I, Berg A, Grathwohl D et al (1999) Freiburger Fragebogen zur körperlichen Aktivität – Entwicklung, Prüfung und Anwendung. Soz.-Präventivmed. 44:55-64

- - - - - - - - -

Craig CL, Marshall AL, Sjostrom M et al (2003) International Physical Activity Questionnaire: 12-country reliability and validity. Medicine and Science in Sports and Exercise 35:1381-1395

Rütten A, Vuillemin A, Ooijendijk W et al (2003) Physical activity monitoring in Europe. The European Physical Activity Surveillance System (EUPASS)

approach and indicator testing. Public Health Nutr. 6 (4):377-84

Mäder U, Martin BW, Schutz Y (2006) Validity of four short physical activity questionnaires in middle-aged persons. Med Sci Sports Exerc. 38 (7):1255-66

Jospeh KL, Dagfinrud H, Christie A et al (2021) Criterion validity of The International Physical Activity Questionnaire-Short Form (IPAQ-SF) for use in clinical practice in patients with osteoarthritis. B;C Musculoskeletal Disorders 2:232

<u>Literature on EHIS-PAC:</u>

Finger JD, Tafforeau J, Gisle L et al (2015) Development of the European Health Interview Survey - Physical Activity Questionnaire (EHIS-PAQ) to monitor physical activity in the European Union. Archives of Public Health 73:59

Robert Koch-Institut (Hrsg.) (2017) Körperliche Bewegung bei der Arbeit, zur Fortbewegung und in der Freizeit. In: Robert Koch-Institut (Hrsg.). Fragebogen zur Studie „Gesundheit in Deutschland aktuell“: GEDA 2014/2015-EHIS. Journal of Health Monitoring 2(1):105–135

Baumeister SE, Ricci C, Kohler S et al (2016) Physical activity surveillance in the European Union: reliability and validity of the European Health Interview Survey-Physical Activity Questionnaire (EHIS-PAQ). International Journal of Behavioral Nutrition and Physical Activity: 13:61

<u>Other literature:</u>

Robert Koch Institue (Berlin), Scientific Institute of Public Health (Brussels), National Institut for Health Development (Estonia). Revised Interim Report EHIS-Project: Improvement of the EHIS modules. Berlin: Robert Koch Institute; 2011.

Bull FC, Bull FC, Maslin TS et al (2009) Global physical activity questionnaire (GPAQ): nine country reliability and validity study. J Phys Act Health 6 (6):790–804

Ainsworth BE, Caspersen CJ, Matthews CE et al (2012) Recommendations to improve the accuracy of estimates of physical activity derived from self report. J Phys Act Health 9(01):76

Savinainen M, Nygard CH, Ilmarinen J. (2004) A 16-year follow-up study of physical capacity in relation to perceived workload among ageing employees. Ergonomics 47(10):1087–102.

Schibye B, Hansen AF, Sogaard K, et al (2001) Aerobic power and muscle strength among young and elderly workers with and without physically

demanding work tasks. Appl Ergon. 32(5):425–31.

Kahlmeier S, Kelly P, Foster C et al (2014) Health economic assessment tools (HEAT) for
walking and for cycling. Methodology and user guide. Economic assessment of transport infrastructure and policies. WHO Regional Office for Europe, Copenhagen

WHO Regional Office for Europe (2014) From Amsterdam to Paris and beyond: the Transport, health and Environment Pan-European Programme (THE PEP) 2009–2020. WHO Regional Office for Europe, Copenhagen

WHO Regional Office for Europe (2014) Physical activity promotion in socially disadvantaged groups: principles for action. WHO Regional Office for Europe, Copenhagen

Construct (genral)	<u>**Health Literacy**</u>
**Short description**	The European Health Literacy Consortium [Sørensen et al., 2012] defines health literacy as follows: "Health literacy is linked to literacy and entails people's knowledge, motivation and competence to access, understand, appraise and apply health information in order to make judgments and take decisions in everyday life concerning healthcare, disease prevention and health promotion to maintain or improve quality of life during the life course." The authors describe an integrated model of health literacy. 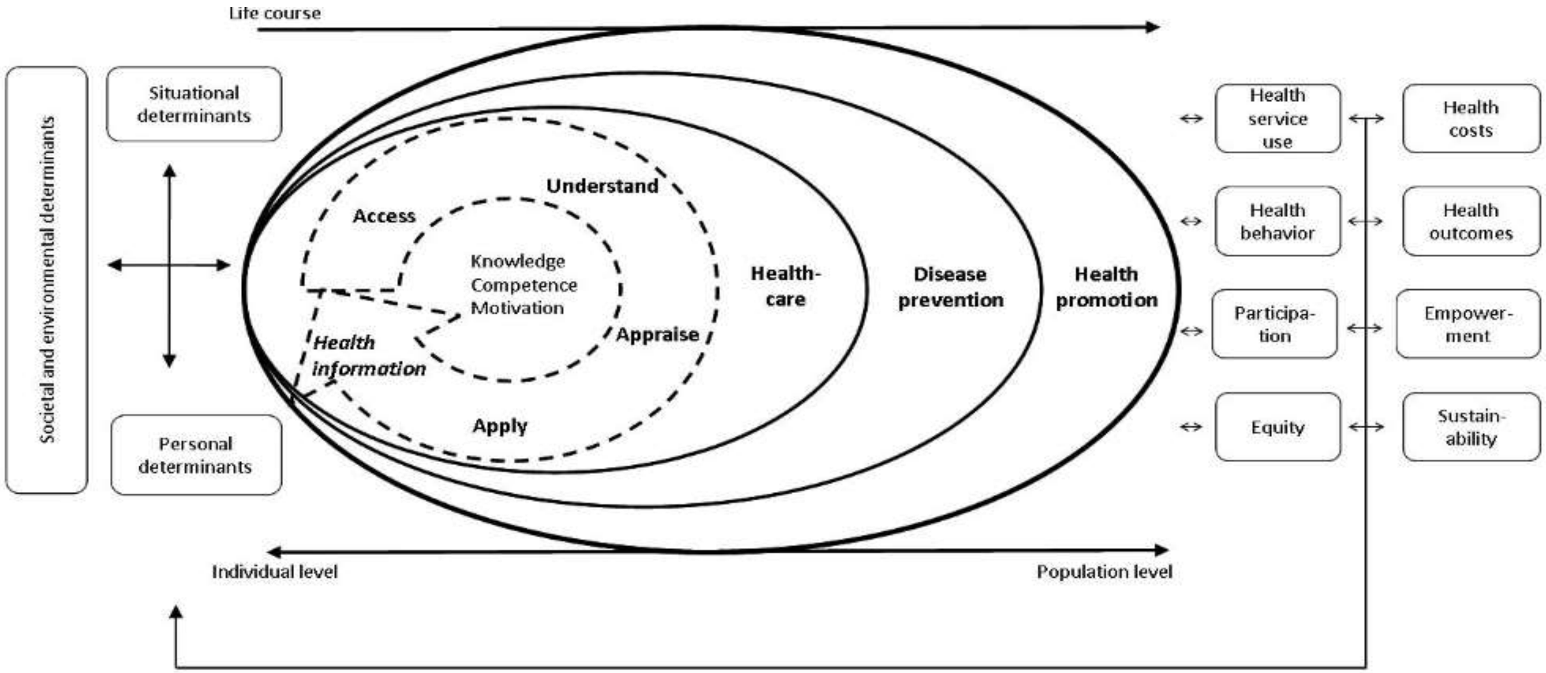 The definition of health literacy centers on four key steps: being able to locate and access health-related information, understand it, assess it, and apply it for oneself or others. Surrounding these central steps are additional facets, such as motivation, intention, and willingness to take responsibility for one's health, assertiveness, the ability to utilize social resources, and navigating the healthcare system.

In Germany, the Federal Ministry of Health established an "Alliance for Health Literacy" in 2017. Participants include entities such as the German Medical Association, the National Association of Statutory Health Insurance Funds (GKV), the Federal Association of Therapists, the German Nursing Council, and many others. The "Alliance for Health Literacy" addresses three overarching themes: (1) increasing health literacy through health education, (2) providing reliable information resources, and (3) improving communication competence among involved stakeholders and patients (“communicative medicine”).

To enhance health literacy across the population, renowned experts developed the “National Action Plan for Health Literacy” [Schaeffer et al., 2018] from a scientific perspective following the establishment of the "Alliance for Health Literacy" in 2018. It includes 15 recommendations across four areas of action: (1) Promoting health literacy in all areas of life, (2) designing a health-literate and user-friendly healthcare system, (3) managing health literacy with chronic illnesses, and (4) systematically researching health literacy.

The HLS-GER2[2] [7] study investigated health literacy in Germany [Schaeffer et al., 2018]. In a study involving 2,151 participants aged 18 and older, paper-assisted personal interviews were conducted between December 2019 and January 2020. The results show that 28.4% of participants had inadequate, 30.4% problematic, 26.5% sufficient, and 14.7% excellent health literacy. Thus, more than half of the population in Germany has problematic or inadequate health literacy. Additionally, the study reveals that the health literacy of the German population has declined in recent years. The most significant challenges are evident in two areas: (1) 74.9% find it (very) difficult to assess health information, and (2) 67.7% have difficulties managing information related to health promotion. Particularly affected groups include individuals with low educational levels (78.3%), low social status (71.9%), older adults (65.1%), and those with chronic pain (62.3%).

The authors, Schaeffer et al. (2018), suggest that individuals with low health literacy are more likely to perceive their health as poor, engage less in health-promoting behaviors, have higher rates of sickness-related absenteeism at work, more frequent doctor visits, more hospital stays, and a higher reliance on emergency medical services.

At the same time, the prevalence of physical inactivity is increasing [Froböse et al., 2018]. For more information on this topic, refer to the construct of physical activity. Sudeck et al. (2022) note that the domain of movement has thus far been largely overlooked in general concepts of health literacy. The authors highlight examples such as the "processing of movement-related cognitive and sensorimotor information with health relevance or understanding and utilizing health-related information during independent physical and sports activity," which remain unaddressed. They further argue that

[7] HLS-GER[2] – Second Health Literacy Survey Germany

these questions are "not trivial when it comes to health-literate evaluations, decisions, and actions to classify the individual relationship between movement, health, and well-being" and to address them purposefully in movement interventions.

Against this backdrop, a domain-specific model of movement-related health literacy (bGK) was developed [Pfeifer et al., 2013; Sudeck and Pfeifer, 2016]. The movement-related health literacy model aims to build "health-effective physical activity" that goes far beyond the previously focused functional improvement or restoration. It emphasizes relevant activities, aspects of participation, and individual contextual factors.

The model assumes that individuals with high bGK are capable of independently incorporating regular health-promoting physical activity into their daily lives. It consists of three components: movement (basic movement-related skills and abilities), control (basic knowledge related to the body and movement), and self-regulation competence (supportive personal action characteristics and attitudes) [Pfeifer et al., 2013; Sudeck and Pfeifer, 2016; Carl et al., 2020b].

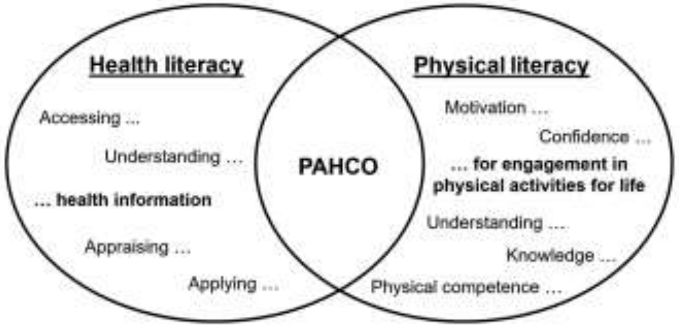

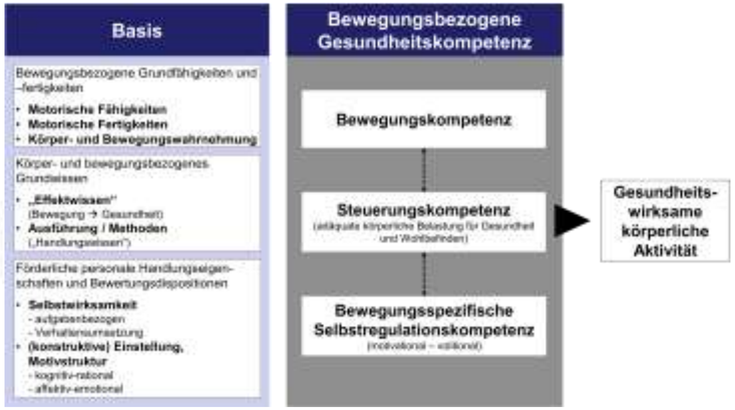

From the experience of pain therapy, a lack of competencies in coping with and preventing illnesses has emerged as a negative influencing factor in the development of chronic pain among chronic or chronified pain patients. This deficiency not only contributes to the persistence of pain but also to the manifestation of psychosocial and somatic impairments that extend beyond the symptom of pain itself. It can be assumed that health literacy is connected to the ability to cope with pain, prevent it proactively, and promote health. The current state of health literacy in Germany, as well as political

	developments such as the "Alliance for Health Literacy" and the "National Action Plan for Health Literacy," also suggest that health literacy is indispensable as a component of the outpatient interdisciplinary multimodal pain therapy (A-IMPT). To evaluate whether health literacy improves through participation in group therapy, an accompanying evaluation was planned, focusing on (1) general health literacy and (2) movement-related health literacy.<br><br>Methods for measuring individual health literacy address the ability and intention of individuals to engage with health information, the healthcare system, and its resources. More than 100 measurement tools are already available in the American linguistic and cultural context [Bitzer and Sørensen, 2018]. These tools differ in several ways, including the mode of administration (e.g., written, telephone, personal interview), their length and duration, their purpose (screening, monitoring, effect evaluation), available languages, and their psychometric quality. Questionnaires also vary in their target groups, such as the HELP tool for individuals with chronic musculoskeletal diseases, FCCHL for diabetes patients, or HLS-NRW-Q for older individuals, people with limited basic education, and those with a migration background.<br><br>In the context of health literacy, differences along the following dimensions are also relevant: facets of health literacy, levels of health literacy, survey design, and content orientation [Jordan et al., 2011; Nguyen et al., 2015; O Neill et al., 2014; Altin et al., 2014; Haun et al., 2014]. The majority of instruments for measuring health literacy focus narrowly on functional health literacy and are designed as individually administered tests. These instruments often have a pronounced exam-like character, which significantly reduces acceptance among respondents and carries a substantial risk of (self-)stigmatization [Kronzer, 2016].<br><br>In recent years, self-assessment questionnaires have gained importance. Approximately 40 self-assessment questionnaires have been scientifically published [O’Neill et al., 2014]. Unlike test-based questionnaires, self-assessment tools are generally broader in scope and aim to capture respondents’ perspectives on aspects such as finding, evaluating, and applying health information.
**Chosen Test**	Questionnaire on individual health literacy
**Authors**	Wieland and Hammes, 2018
**Rationale for the selection:**	The test was chosen based on face validity, not because of its background in workplace health promotion. Alternative questionnaires were often extensive in terms of the number of items, so for resource reasons, the decision was made to use this particular questionnaire.

**Short description**	The questionnaire on individual health literacy consists of 10 items, which patients rate on a 5-point scale ranging from "does not apply at all" to "applies completely." The underlying concept of the questionnaire is composed of three dimensions of health literacy: "Health Goals," "Confidence in Success," and "Coping Ability." The questionnaire captures individual experiences, expectations, and the ability to actively and effectively address health complaints and illnesses, as well as to maintain and promote health through appropriate measures. To date, the questionnaire has primarily been used in the context of workplace health promotion.<br>According to the authors, the construct of health literacy is closely linked to the concept of self-efficacy as described by Bandura, O'Leary, and Schwarzer.
**Scales (Items)**	10 Items
**Response format**	5-point scale from “not at all true” to “completely true”
**Standard values**	/
**Test psychometrics**	*see below*
Content Validity	No information is available.
Construct Validity – Convergent Validity	No information is available.
**Criterion Validity**	No information is available.
Reliability – Internal Consistence	Cronbachs Alpha: 0,7 (Wieland und Hammes, 2008)
**Change sensitivity**	In 3 studies, a significant change in health literacy in the intervention groups was demonstrated -> sensitivity to change given (Wieland & Latocha, 2015)
**Test criticism**	So far, no feedback from the authors upon request, so statements regarding the test psychometrics are not possible. The actual development process of the questionnaire is therefore not traceable at this point in time. (Wieland, R. & Hammes, M. (in preparation). Questionnaire on Health Literacy (GKF) – Theoretical Foundations and Empirical Validation.)
**Chosen Test**	Questionnaire for assessing movement-related health literacy (BGK)

**Authors**	Sudeck and Pfeifer, 2016
**Rationale for the selection:**	There is no other questionnaire to assess movement-related health literacy.<br><br><u>Adjustments for the project:</u><br>The long version with 42 items is too long, and the short version with 14 items is too short and not validated. In consultation with the author team and due to resource considerations as well as practicality, the questionnaire was reduced to address the relevant aspects within the framework of A-IMPT.
**Short description**	The questionnaire for assessing movement-related health literacy measures cognitive and motor skills required for engaging in health-promoting physical activity, as well as the associated motivational, volitional, and social readiness/abilities for the successful and responsible integration of health-promoting physical or sporting activity into various situations in daily life. The questionnaire consists of 42 items and is recommended to be used in combination with the BSA (see above).
**Scales (Items)**	42 items, short version (not validated) 16 items; selected items from the three scales are used in PAIN2.0
**Response format**	varies depending on the sub-area/question
**Standard values**	/
**Test psychometrics**	
Content Validity	No information is available.
Reliability – Internal Consistence	*Sample: Study A=1057; Study B=3603*<br><br>Movement competence: α 0,88/0,89 (Pfeifer and Sudeck, 2016)<br>Control competence: α 0,80/0,84 (Pfeifer and Sudeck, 2016)<br>Self-regulation competence: α 0,78/0,80 (Pfeifer and Sudeck, 2016)
Indicator reliability	Factor loadings for all indicators of the three factors were statistically significant ($p < .05$) and can generally be assessed as satisfactory to good (see Fig.). (Pfeifer and Sudeck, 2016)

	Anomalies in the Squared Multiple Correlations: Anomalies in the area of control competence for physical training (CPT): Indicators CPT3 and CPT5, whose measurement error covariance was included in the model, fell below the recommended threshold (each SMC = 0.28 ≤ 0.40). (Pfeifer and Sudeck, 2016)<br>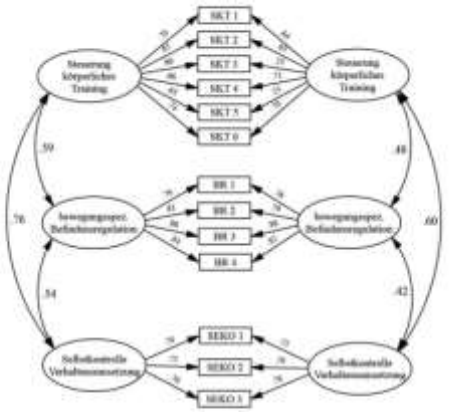
Construct Validity – Convergent Validity	To assess convergent validity, the following were used: construct reliability (H) and the average extracted variance (AEV):<br>For all three factors (see above), both in Study A (CPT: H = .83; BR [movement-specific mood regulation]: H = .89; SEKO [self-control for behavioral implementation]: H = .78) and in Study B (CPT: H = .79; BR: H = .88; SEKO: H = .80), good to very good factor reliabilities were found, with H values > 0.60 recommended. While the average extracted variance for self-control (Study A: AEV = .54; Study B: AEV = .57) and mood regulation (Study A: AEV = .67; Study B: AEV = .65) was good (AEV ≥ 0.50), this indicator for control competence in physical training (Study A: AEV = .46; Study B: AEV = .40) fell below the threshold. Therefore, the convergent validity for self-control and mood regulation was considered good, while mixed results were found for control competence in terms of the two criteria H and AEV. (Pfeifer and Sudeck, 2016)
Construct Validity – Discriminant Validity	To assess discriminant validity, the criterion proposed by Fornell and Larcker was used, according to which the average extracted variance (AEV) of a factor should be greater than its squared correlation with other factors in the model: For discriminant validity, no limitations were found for the factors of self-control and mood regulation. For these two latent factors, the lowest correlations were found in both studies (see figure for indicator reliability; Study A: r = .54; Study

	B: r = .42). In contrast, for control competence in physical training, the Fornell-Larcker criterion was violated in Study A. The AEV was lower than the squared factor correlation between control competence and self-control (AEV = 0.46 < $(0.76)^2$ = 0.58). However, this limitation in discriminant validity was not confirmed by Study B, which showed overall lower factor correlations. Despite the lower AEV for control competence, it was higher than the respective squared factor correlations. (Pfeifer and Sudeck, 2016)
**Change sensitivity**	Change sensitivity for patients with COPD given (Carl und Geidl et al., 2021)
**Test criticism**	/
**Test psychometrics of the underlying PAHCO model of physical activity-related health literacy**	

The model of physical activity-related health literacy (PAHCO) offers an integrative and interdisciplinary view on the competencies necessary for individuals who wish to maintain a healthy, physically active lifestyle. Given the need to collect further potential evidence for the validity of this model through the development of an assessment instrument, the aim of the study by Carl et al. (2020) was to expand initial measurement models for PAHCO.

Method: In Study 1, a measurement model with five predictors for PAHCO was tested on 341 COPD patients undergoing inpatient rehabilitation. In Study 2, data from 745 trainees were used to create an expanded eight-factor measurement model. To assess the validity of the models, a reliability analysis, confirmatory factor analysis (CFA), and structural equation modeling (SEM) were performed.

The analyses yielded good results for the reliability and discriminant validity of the factors. Accordingly, the CFA showed satisfactory model fits for both the five-factor and expanded eight-factor models. Associations with physical activity and physical health parameters indicated criterion validity for seven of the eight PAHCO factors. The explained variance of the multivariate models ranged between 9.8% and 10.4% in Study 1 and between 9.5% and 21.3% in Study 2. It was therefore possible to extract a well-fitting eight-factor measurement model and gather additional evidence for the validity of the PAHCO model (Carl et al., 2020).

**Further tests in the shortlist that were excluded:**	
**HLS-EU-Q[8]**	
**Authors**	[Sørensen et al. 2012]

[8] European Health Literacy Survey Questionnaire

**Short description**	The HLS-EU-Q assesses the subjectively perceived difficulties in managing health-related information. It considers how a system, organization, or profession enables or hinders users in finding, understanding, assessing, and applying (areas of action of health literacy) health information within the context of healthcare, disease prevention, and health promotion. The HLS-EU-Q was developed and applied as part of the HLS-EU study. It is a self-assessment tool consisting of 47 items. The items are based on the definition of health literacy by Sørensen et al. (2012) and the associated conceptual model. There is also a short form with 16 items. The questions are answered on a four-point scale: very easy, fairly easy, fairly difficult, very difficult. The original version was written in English. To apply the questionnaire in the participating European countries as part of the HLS-EU study, it was generally translated into German. The German-language questionnaire has already been used in several studies (see Pelikan et al., 2019; includes studies by Jordan et al., 2015; Berens et al., 2016; Schäfer et al., 2016 and 2017a; Vogt et al., 2017).
**Psychometric properties**	No psychometric properties have been established for the German-language questionnaire so far.<br>In other countries, however, the HLS-EU-Q has already been examined for its psychometric properties and has generally been assessed as valid and reliable, for example:<br>Greece: Michou and Costarelli (2022)<br>Sweden: Bergman et al. (2023)<br>Arabia: Bergman et al. (2023)<br>Slovenia: Japelj and Horvat (2022)
**Exclusion reasons**	The conceptual model described above by Sørensen et al. (2012) has only been partially incorporated into the A-IMPT (specifically understanding and evaluating; the areas of finding and appraising are addressed to a lesser extent).<br>Since the design of the A-IMPT does not fully represent the conceptual model and the questionnaire covers too broad a scope, it was not selected.
**Health Literacy Questionnaire (HLQ)**	
**Authors**	Osborne et al., 2013
**Short description**	The HLQ was originally developed in 2012 and is now used in many countries and languages as an instrument to measure health literacy. It is a tool that derives results based on respondents' self-assessment. The German-language version has been available since 2017. The HLQ-G consists of 44 items designed to measure the health literacy of

	adults. The 44 items are divided into 9 domains, each containing either 4, 5, or 6 items. These domains include, among others: feeling understood and supported by healthcare providers, actively managing one's health, or appraising health information. The questions in 5 domains can be rated on a 4-point Likert scale ("strongly disagree" – "disagree" – "agree" – "strongly agree"). The questions in the remaining 4 domains can be rated on a 5-point Likert scale ("cannot do" to "very easy").
**Psychometric properties**	The original development of the Health Literacy Questionnaire (HLQ) was conducted by Osborne et al. (2013). Using a validity-driven approach, broad and conceptually distinct domains were identified through workshops and interviews. The questionnaire items were directly derived from consultation data, adhering to a rigorous process to capture the full range of experiences from individuals currently active in healthcare to those in the general population. Psychometric analyses included confirmatory factor analysis (CFA) and item response theory. Cognitive interviews were conducted to ensure that the questions were understood as intended. Initially, the items were tested on a calibration sample consisting of participants from healthcare settings, home care, and hospitals (N=634), and subsequently on a replication sample (N=405) comprising individuals who had recently visited an emergency department.<br><br>A total of 91 items were originally developed, distributed across 6 scales with "agree/disagree" response options and 5 scales with "difficulty performing tasks" response options. Cognitive testing revealed that most items were well-understood, requiring only minor rewording. Psychometric testing of the calibration sample identified 34 poorly performing or conceptually redundant items, which were removed, resulting in 10 scales. These were then tested and refined in a replication sample, leading to 9 final scales with 44 items. A 9-factor CFA model was fitted to these items, allowing no cross-loadings or correlated residuals. Despite the highly constrained nature of the model, the fit was satisfactory: χ^2WLSMV(866 d.f.) = 2927, p<0.000, CFI = 0.936, TLI = 0.930, RMSEA = 0.076, and WRMR = 1.698.<br><br>The final scales included: Feeling understood and supported by healthcare providers, Having sufficient information to manage my health, Actively managing my health, Social support for health, Appraising health information, Ability to actively engage with healthcare providers, Navigating the healthcare system, Ability to find good health information, and Understanding health information well enough to know what to do.<br><br>The HLQ captures 9 conceptually distinct domains of health literacy to address the needs and challenges of a broad range of individuals and organizations. Given its validity-driven approach, the HLQ is likely to be valuable in surveys, intervention evaluations, and studies exploring the needs and capabilities of individuals.<br><br>German-language version (n=1074):

	Validity: Confirmatory Factor Analysis (CFA) – $\chi^2SB(866) = 2948.1$, $p>0.000$<br>Root Mean Square Error of Approximation (RMSEA): 0.048 (90% CI: 0.046; 0.050)<br>Non-Normed Fit Index (NNFI): 0.99<br>Comparative Fit Index (CFI): 0.99<br>Standardized Root Mean Residual (SRMR): 0.075<br>*Reliability: Internal consistency* – Cronbach's alpha ranging from 0.77 to 0.91, depending on the domain [Nolte et al., 2017].<br>*Change Sensitivity*: Not investigated.
**Exclusion reasons**	The literature recommends using the questionnaire, particularly for improving services and developing interventions [Nolte et al., 2017]. However, this is only partially applicable to the context of the A-IMPT in PAIN2.0, which is why the questionnaire was excluded.

Citations

Literature on the *construct of health literacy*:

Altin S, Finke I, Kautz-Freimuth S et al (2014) The evolution of health literacy assessment tools: A systematic review. BMC Public Health 14:1207.

Bitzer E-M, Sørensen K (2018) Gesundheitskompetenz – Health Literacy. Gesundheitswesen 80:754-766

Bundesministerium für Gesundheit (2017). Allianz für Gesundheitskompetenz. Gemeinsame Erklärung. https://www.bundesgesundheitsministerium.de/fileadmin/Dateien/3_Downloads/E/Erklaerungen/Allianz_fuer_Gesundheitskompetenz_Abschlusserklaerung.pdf. Zugegriffen: 20.10.2023

Carl J, Sudeck G, Pfeifer K (2020a) Competencies for a healthy physically active lifestyle – reflections on the model of physical activity-related health competence. J Phys Act Health 17:688–697

Froböse I, Biallas B, Walmann-Sperlich B (2018) Der DKV-Report 2018 „Wie gesund lebt Deutschland?“. DKV Deutsche Krankenversicherung, Düsseldorf

Haun JN, Valerio MA, McCormack LA et al (2014) Health literacy measurement: an inventory and descriptive summary of 51 instruments. Journal of Health Communication 19 (Suppl 2):302–333

Jordan JE, Osborne RH, Buchbinder R. (2011) Critical appraisal of health literacy indices revealed variable underlying constructs, narrow content and psychometric weaknesses. J Clin Epidemiol 64:366–379

Kronzer VL (2016) Screening for health literacy is not the answer. BMJ (Clinical research ed.) 354: i3699

Nguyen TH, Park H, Han H-R et al (2015) State of the science of health literacy measures: Validity implications for minority populations. Patient Education and Counseling 98:1492–1512

O Neill B, Gonçalves D, Ricci-Cabello I et al (2014) An overview of self-administered health literacy instruments. PLoS ONE 9:e109110

Pfeifer K, Sudeck G, Geidl W, Tallner A (2013) Bewegungsförderung und Sport in der Neurologie – Kompetenzorientierung und Nachhaltigkeit. Neurol Rehabil 19:7–19

Schaeffer D, Berens EM., Gille S et al (2021) Gesundheitskompetenz der Bevölkerung in Deutschland – vor und während der Corona Pandemie: Ergebnisse des HLS-GER 2. Bielefeld: Interdisziplinäres Zentrum für Gesundheitskompetenzforschung (IZGK), Universität Bielefeld. DOI: https://doi.org/10.4119/unibi/2950305

Schaeffer D, Hurrelmann K, Bauer, U et al (2018) Nationaler Aktionsplan Gesundheitskompetenz. Die Gesundheitskompetenz in Deutschland stärken. KomPart: Berlin

Sørensen K, van den Broucke S, Fullam J et al (2012) Health literacy and public health: A systematic review and integration of definitions and models. BMC Public Health 12:1–13

Sudeck G, Rosenstiel S, Carl J. et al (2022) Bewegungsbezogene Gesundheitskompetenz – Konzeption und Anwendung in Gesundheitsförderung, Prävention und Rehabilitation. In: Rathmann K et al (Hrsg.) Gesundheitskompetenz, Springer Reference Pflege – Therapie – Gesundheit, Gesundheit https://doi.org/10.1007/978-3-662-62800-3_135-1

Sudeck G, Pfeifer K (2016) Physical activity-related health competence as an integrative objective in exercise therapy and health sports – conception and validation of a short questionnaire. Sportwissenschaft 46:74–87

Literature on *the available questionnaires*:

Sorensen K, Van den Broucke S, Pelikan J et al (2013) Measuring health literacy in populations: illuminating the design and development process of HLS-EU-Q. BMC Public Health 13:948

Pelikan J.M., Ganahl K., Borucke van den S et al (2019) Measuring health literacy in Europe: Introducing the European Health Literacy Survey Questionnaire (HLS-EU-Q). In: Okon et al (Hrsg.) International Handbook of Health Literacy. Research, practice and policy across the lifespan. Policy Press: Bristol UK, https://doi.org/10.51952/9781447344520.ch008

Jordan S and Hoebel J (2015) 'Gesundheitskompetenz von Erwachsenen in Deutschland. Ergebnisse der Studie „Gesundheit in Deutschland aktuell" (GEDA). Bundesgesundheitsblatt Gesundheitsforschung Gesundheitsschutz 58 (9):942-50

Berens EV, Vogt D, Messer M et al (2016) 'Health literacy among different age groups in Germany: Results of a cross-sectional survey. BMC Public Health 16(1):1151

Schaeffer D, Berens EM and Vogt D (2017a) Health literacy in the German population. Deutsches Ärzteblatt International 114(4):53-60

Schaeffer D, Vogt D, Berens EM et al (2016) Gesundheitskompetenz der Bevölkerung in Deutschland. Ergebnisbericht. Universität Bielefeld, Fakultät für Gesundheitswissenschaften: Bielefeld

Vogt D, Schaeffer D, Messer M, et al (2017) Health literacy in old age: Results of a German cross-sectional study. Health Promotion International 33(5):739-47

Michou M, Vassiliki C (2022) Validity and reliability of the European health literacy survey questionnaire (HLS-EU_Q16) in the greek language. Mediterranean Journal of Nutrition and metabolism 15(2):285-294

Bergman L, Nilsson U, Dahlberg K et al (2023) Validity and reliability of the swedish versions of the HLS-EU-Q16 and HLS-EU-Q6 questionnaires. BMC Public Health 23:724

Bergman L, Nilsson U, Dahlberg K et al (2023) Validity and reliability of the arabic versions of the HLS-EU-Q16 and HLS-EU-Q6 questionnaires. BMC Public Health 23:304

Japelj N, Horvat N (2023) Translation and validation of the European Health Literacy Survey Questionnaire (HLS-EU-Q47) into the Slovenian language. International Journal of Clinical Pharmacy 45(1):387-1395

- - - - - - - -

Osborne RH, Batterham RW, Elsworth GR et al (2013) The grounded psychometric development and initial validation of the Health Literacy

Questionnaire (HLQ). BMC Public Health 13:658

Nolte S, Osborne RH, Dwinger S et al (2017) German translation, cultural adaption and validation of the Health Literacy Questionnaire (HLQ). PLoS ONE, 12(2):e0172340

Literature *selected questionnaires:*

Wieland R, Latocha K (2015) Betriebliche Gesundheitsförderung bei psychisch erkrankten Beschäftigten. Fehlzeiten-Report 2015. Springer, Berlin, Heidelberg, S 313-324

Wieland R, Hammes M (2008) Gesundheitskompetenz als personale Ressource. In: Mozygemba et al (Hrsg.) Nutzenorientierung – ein Fremdwort in der Gesundheitssicherung? Huber, Bern, S 177-190

Sudeck G, Pfeifer K (2016) Physical activity-related health competence as an integrative objective in exercise therapy and health sports – conception and validation of a short questionnaire. Sportwissenschaft 46:74–87

Carl J, Sudeck G, Geidl W et al (2020b) Competencies for a healthy physically active lifestyle – validation of an integrative model. Res Q Exerc Sport. https://doi.org/10.1080/02701367.2020. 1752885

Carl J, Geidl W, Schuler M et al (2021) Towards a better understanding of physical activity in people with COPD: predicting physical activity after pulmonary rehabilitation using an integrative competence model. Chronic Respiratory Disease 18:1-12

Therapy expectancy

**Construct (general)**	**Therapy expectancy / Therapy motivation (we assessed the therapy expectation)**
**Short description**	The expectations, hopes, and fears of a patient influence the therapeutic process. They can affect both the course and outcome of therapy (Barker, Funk, and Houston, 2008; Stevens, Hynan & Allen, 2000), although historically their impact has been underestimated (Edward & Fehm, 2023). Today, therapy expectations are generally regarded as potential active factors that can influence the success of therapeutic approaches (Edward & Fehm, 2023). Without confidence in the effectiveness of therapy or with fears regarding unwanted side effects, patients' motivation and engagement in therapy decrease (Schulte & Eifert, 2002). The significance of placebo and nocebo effects has been demonstrated in numerous studies (e.g., review by Colloca & Barsky, 2020), making it particularly relevant to consider therapy expectations in evaluations, especially in samples of pain patients. The impact of positive expectations on therapeutic success has been examined in numerous studies, showing a small but significant correlation between more positive

	expectations and reduced symptom burden at the end of treatment (r = .18, p < .001, d = .36; Constantino, Vîslă, Coyne & Boswell, 2018).<br>Therapy expectancy is defined as patients' assumptions about the consequences of participating in a treatment. Therapy motivation, which describes the desire and willingness for change, does not necessarily align with positive therapy expectations. Therapy credibility is a separate construct that focuses more on the perception of an intervention during its execution (Constantino et al., 2011). In this study, we focus on the construct of therapy expectations.<br>Therapy expectations are among the nonspecific patient-related factors that can influence the course and outcome of psychotherapy (Schulte, 2005). In addition to positive outcome expectations, fears about unwanted consequences of therapy can also play a role. The latter, for example, is relevant in anxiety treatment (Reiss & McNally, 1985). In a study by Edwards and Fehm (2023), both positive and negative expectations of patients were examined as factors using the "Patient Questionnaire on Therapy Expectation and Evaluation" (PATHEV). For this purpose, the subscales "Hope for Improvement" and "Fear of Change" of the PATHEV were used and analyzed as additional predictors for individual therapy success. Alongside established success factors such as symptom burden at the start of therapy and the number of diagnoses, therapy expectations appear to be an additional predictor of symptom remission. Edwards and Fehm (2023) therefore recommend the routine assessment of therapy expectations at the beginning of therapy.
**Chosen Test**	PATHEV (Patient Questionnaire on Therapy Expectations and Evaluation)
**Author**	[Schulte, 2005]
**Rationale for the selection:**	We decided to use the PATHEV for the assessment of therapy expectations because it most closely captures what we wanted to measure in terms of (apparent) validity. Particularly, the aspect of pessimistic outcome expectation/skepticism is absent in most other questionnaires, and many specific questionnaires are focused either on particular treatment contexts (e.g., rehabilitation) or diseases (e.g., anxiety disorders, such as the Reaction to Treatment Questionnaire, RTQ, Holt & Heimberg, 1990) (Constantino et al., 2011). In three studies on construct validity, the scales were found to be theory-conform. They allow for the prediction of 13-17% of therapy success (Schulte, 2005). The internal reliability (Cronbach's alpha, see below) is sufficiently good for all scales, ranging from approximately .7 to .8. With 11 items, the argument for the economy of this questionnaire was particularly strong.
**Short description**	The "Patient Questionnaire on Therapy Expectations and Evaluation (PATHEV)" was developed based on two self-constructed questionnaires on the psychotherapy process, which have been used in this form by Schulte since 1996:

	the "Questionnaire on Patient Expectations (PATERWA 96)" with four factor-analytically derived subscales: "Hope for Improvement (Confidence)," "Fear of Change," "Hope for Social Improvement (Social Pressure)," and "Fear of Social Discrimination," and the "Questionnaire for Therapy Evaluation (THEBEWE 96)" with four subscales: "Passivity/Role Expectations," "Importance of Therapy," "Belief in Therapy," and "Doubts about Therapy." The PATERWA subscales "Hope for Improvement" and "Fear of Change" correspond to the patient's expectation of therapy outcomes, and the THEBEWE subscales "Belief in Therapy" and "Doubts about Therapy" were designed to capture the degree of fit. The resulting PATHEV consists of three subscales: Hope for Improvement, Fear of Change, and Fit (Schulte, 2005).
**Scales (Items)**	The PATHEV consists of 11 items based on 3 subscales: "Hope for Improvement," "Fear of Change," and "Fit."
**Response format**	Patients are asked to rate each item on a scale from 1 (strongly disagree) to 5 (strongly agree).
**Standard values**	Not found
**Test psychometrics** [9]	
Content Validity	not investigated
Construct Validity – Convergent Validity	"In three studies on construct validity, the scales were found to be theory-consistent; they allow for the prediction of 13% and 16% of the variance in therapy outcomes." (Schulte, 2005)
**Criterion Validity**	not investigated
Reliability	**Hope for improvement** Cronbach´s Alpha = 0,89<br>**Fear of Change** Cronbach´s Alpha = 0,73<br>**Fit** Cronbach´s Alpha = 0,82
**Change sensitivity**	not investigated
**Test criticism**	Unknown
**Further tests in the shortlist that were excluded:**	
**FPTM-23** (Questionnaire on Psychotherapy Motivation)	
**Author**	[Schulz et al., 1995]

[9] Based on the COSMIN specifications, see below the overall table

**Short description**	The Psychotherapy Motivation Questionnaire (FPTM) assesses psychotherapy motivation in the rehabilitation context. It consists of the following scales: Psychological Distress ("I suffer greatly from emotional problems"), Hope ("I am optimistic about the improvement of my symptoms"), Denial of Psychological Need for Help ("Not being able to cope with emotional stress is a clear sign of weakness"), Knowledge ("I have thoroughly informed myself about the treatment here"), Initiative ("I have actively sought to receive treatment here"), and Symptom-Related Attention from Others ("When I have symptoms, people around me are more understanding than usual").
**Psychometric properties**	*Validity:*<br>The validity was examined through factorial structure, external ratings, use of psychotherapeutic services, as well as short- and long-term treatment outcomes, with – except for a few minor exceptions – generally satisfactory to good theory-conforming relationships found.<br>*Change Sensitivity:* not investigated<br>*Reliability (internal consistency):* Satisfactory.<br>*Psychological distress and self-reflection*: Cronbach's alpha = 0.90<br>*Hope*: Cronbach's alpha = 0.81<br>*Denial of psychological need for help*: Cronbach's alpha = 0.76<br>*Knowledge*: Cronbach's alpha = 0.71<br>*Initiative:* Cronbach's alpha = 0.86<br>*Symptom-related attention from others:* Cronbach's alpha = 0.89
**Exclusion reasons**	The FPTM was excluded due to issues of validity (less apparently valid, as it measures therapy motivation rather than expectations) and economy (39 items).
**PAREMO** (Patient Questionnaire for Assessing Rehabilitation Motivation)	
**Authors**	[Nübling et al., 2005]
**Short description**	The Patient Questionnaire for Assessing Rehabilitation Motivation (PAREMO) assesses aspects of rehabilitation motivation through six scales. It can be used in rehabilitation diagnostics, treatment planning, and research.<br>Scales: Psychological distress, Body-related limitations, Social support and illness gain, Willingness to change, Knowledge regarding rehabilitation measures, Skepticism.
**Psychometric properties**	Standard values available for patients in rehabilitation

	Validity:<br>*Construct Validity*<br>Correlations were investigated with psychotherapy motivation, life satisfaction, social support, depression, anxiety, rehabilitation-specific expectations, subjective health status, as well as with the external assessment of motivation by the doctor/therapist and questions from the intake documentation. However, the validation results also show that particularly for the scales "Willingness to Change," "Information Regarding Rehabilitation," and "Skepticism," validity could not be demonstrated or was insufficient.<br><br>*Reliability – Internal Consistency – Cronbach's Alpha*<br>Varies between 0.67-0.91 across the scales<br><br>*Change Sensitivity*: not investigated
**Exclusion reasons**	The PAREMO was not selected due to reasons of validity (face validity, especially for the rehabilitation context) and economy (20 items).

Citations

Literature on the construct of therapy expectation:

Barker, S. L., Funk, S. C., & Houston, B. K. (1988). Psychological treatment versus nonspecific factors: A meta-analysis of conditions that engender comparable expectations for improvement. *Clinical Psychology Review*, *8*(6), 579-594.

Colloca, L., & Barsky, A. J. (2020). Placebo and nocebo effects. *New England Journal of Medicine*, *382*(6), 554-561.

Constantino, M. J., Arnkoff, D. B., Glass, C. R., Ametrano, R. M., & Smith, J. Z. (2011). Expectations. *Journal of clinical psychology*, *67*(2), 184-192.

Constantino, M. J., Vîslă, A., Coyne, A. E. & Boswell, J. F. (2018). A meta-analysis of the association between patients' early treatment outcome expectation and their posttreatment outcomes. Psychotherapy (Chicago, Ill.), 55(4), 473 – 485. https://doi.org/10.1037/pst0000169

Edwards, L., & Fehm, L. (2023). Positive und negative Therapieerwartungen sagen Therapieerfolg vorher. *Zeitschrift für Klinische Psychologie und Psychotherapie*.

Grencavage, L. M. & Norcross, J. C. (1990). Where are the commonalities among the therapeutic common factors.

Professional Psychology: Research and Practice, 21, 372–378.

Reiss, S. & McNally, R. J. (1985). Expectancy model of fear. In S. Reiss & R. R. Bootzin (Eds.), *Theoretical issues in behavior therapy* (pp. 107–121). San Diego: Academic Press.

Schulte, D. (2005). Messung der Therapieerwartung und Therapieevaluation von Patienten (PATHEV). Zeitschrift für Klinische Psychologie und Psychotherapie, 34, 176-188.

Schulte, D. & Eifert, G. H. (2002). What to do when Manuals Fail? The Dual Model of Psychotherapy. *Clinical Psychology: Science and Practice, 9*, 312-328.

Stevens, S. E., Hynan, M. T. & Allen, M. (2000). A metaanalysis of common factor and specific treatment effects across the outcome domains of the phase model of psychotherapy. *Clinical Psychology: Science and Practice, 7*, 273–290.

<u>Literature on the available questionnaires:</u>

Berking, M., & Kowalsky, J. (2012). Therapiemotivation. *Klinische Psychologie und Psychotherapie für Bachelor: Band II: Therapieverfahren Lesen, Hören, Lernen im Web*, 13-22.

Constantino, M. J., Arnkoff, D. B., Glass, C. R., Ametrano, R. M., & Smith, J. Z. (2011). Expectations. *Journal of clinical psychology*, *67*(2), 184-192.

Nübling, R., Kriz, D., Herwig, J., Wirtz, M., Fuchs, S., Hafen, K., ... & Bengel, J. (2005). Patientenfragebogen zur Erfassung der Reha-Motivation (PAREMO-20). *Freiburg/Bad Säckingen: Rehabilitationswissenschaftlicher Forschungsverbund (RFV)*.

Nübling, R., Schulz, H., Schmidt, J., Koch, U., & Wittmann, W. W. (2006). Fragebogen zur Psychotherapiemotivation (FPTM)–Testkonstruktion und Gütekriterien. *Reha-Motivation und Behandlungserwartung. Regensburg: Roderer*, 252-270.

Schulte, D. (2005). Messung der Therapieerwartung und Therapieevaluation von Patienten (PATHEV). Zeitschrift für Klinische Psychologie und Psychotherapie, 34, 176-188.

Schulz, H., Lang, K., Nübling, R., & Koch, U. (2003). Psychometrische Überprüfung einer Kurzform des Fragebogens zur Psychotherapiemotivation-FPTM-23. *Diagnostica*, *49*(2), 83-93.

Construct (general)	<u>**Physical Well-Being**</u>
**Short description**	Both in health research and in intervention practice, the construct of "physical well-being" is gaining increasing significance. As a core competence, the ability to generate, experience, and enjoy one's own well-being is considered essential for mastering life in a satisfactory way [Kolip, P., & Schmidt, B., 2006]. "Physical well-being" is seen as an indicator for describing health status and its changes [Kolip, P., & Schmidt, B., 2006]. Thus, the description of health status is no longer defined solely by physical condition and functionality but is also associated with mental state (particularly well-being), highlighting the importance of operationalizing well-being as a construct [Frank, R., 2007].<br><br>As early as 1946, the World Health Organization (WHO) described health as a state of complete physical, mental, and social well-being, and not merely the absence of disease or infirmity [WHO, 1946]. This subjective component of the WHO's definition of health further underscores the central importance of the construct of "physical well-being" as an indirect indicator of health.<br><br>Another foundation is provided by the theory of "embodiment," which describes the interaction between body and mind. This theory highlights the connections between psychological, neurological, body-therapeutic, and systemic/cognitive science fields [Mommert-Jauch, P., 2022]. Its goal is to generate and demonstrate changes in physical states through altered knowledge in patients. Clinical practitioners also point out the high clinical-therapeutic and diagnostic relevance of measuring and assessing "physical well-being," which extends from movement therapy—where patients express various aspects of their physicality—to the perception of movement dynamics and processes of tension and relaxation [FBK-20; Clement & Löwe, 1996].<br><br>Physical well-being is thus regarded as a central dimension of health, but it does not necessarily align with objective health criteria (e.g., physical functional tests, physician assessments) [Frank, R., 2011]. The construct remains relatively heterogeneous in its operationalization, and well-being research predominantly focuses on "psychological well-being,"

even though "physical well-being" is of central value, especially in intervention and rehabilitation research [Schumacher, J., Klaiberg, A., & Brähler, E., 2003].

To clearly define the concept of well-being and its structure, various dimensions are distinguished. The first distinction differentiates between habitual and current well-being [Kolip, P., & Schmidt, B., 2006]:

- **Habitual well-being**: This describes a relatively stable and typical trait of a person, based on aggregated emotional experiences that span weeks and months. Habitual well-being is influenced by both relatively stable personality traits and environmental conditions.
- **Current well-being:** This refers to the momentary, positively tinged state that may be of short duration.

Further differentiation involves breaking well-being into components [Kolip, P., & Schmidt, B., 2006]:

- **Cognitive well-being component**: This represents the subjective evaluation of one's life in terms of individual life satisfaction. It is the result of cognitive processes and relies on personal assessments of one's own life situation.
- **Affective well-being component**: Well-being is described based on current positive and negative feelings and moods.

A third distinction is between one-dimensional and multidimensional constructs. The most well-known multidimensional construct is the WHO model mentioned earlier, which differentiates between physical, mental, and social components of well-being [Kolip, P., & Schmidt, B., 2006].

One challenge is that physical well-being is a subjective phenomenon involving sensory stimuli (sight, hearing, touch/feeling, smell, taste) and interoceptive stimuli (sensations within the body, such as body temperature, visceral and genital sensations, balance). These elicit physical sensations that are perceived and evaluated individually in various body parts or the entire body. The construct of physical well-being, therefore, is not synonymous with physical health or fitness and is not limited to the experience of physical functionality or performance [Frank, R., 2007].

Another difficulty lies in distinguishing physical well-being from the construct of "quality of life." The latter is primarily used in social sciences and medicine and is sometimes used synonymously with "well-being" or is predominantly defined

	by aspects of well-being [Schumacher, J., Klaiberg, A., & Brähler, E., 2003]. Additionally, instruments specifically developed for rehabilitation often aim to measure "quality of life" rather than "well-being" [Kolip, P., & Schmidt, B., 2006].<br><br>In conclusion, no instrument currently exists to measure habitual physical well-being as a relatively stable trait. Existing instruments either focus on psychological well-being or operationalize physical well-being as the absence of complaints, often in the form of physical functional limitations [Kolip, P., & Schmidt, B., 2006].
**Chosen Test**	Based on the above explanation, no questionnaire was selected for the construct of "physical well-being."<br>Another point is that aspects of this parameter are already included in the FW-7 from the DSF and are covered by this instrument.
**FAW** *questionnaire on current physical well-being*	
**Author**	[Frank, R., 2011]
**Short description**	The only instrument that measures current physical well-being and closely aligns with the objective is the "Questionnaire for Assessing Current Physical Well-Being" (FAW) by Frank, R., developed in 2011. The FAW exclusively captures positive aspects of physical well-being [Frank, R., 2007].<br><br>Thus, the FAW is designed to assess current physical well-being, for example, after physical activity or relaxation. It primarily reflects the effects of short-term interventions [Kolip, P. & Schmidt, B., 2006].<br><br>The FAW, in its 58-item version, includes the following factors:<br>1. Satisfaction with the current physical state,<br>2. Calmness/Leisure,<br>3. Vitality/Joy of Life,<br>4. Reduction of Tension,<br>5. Pleasure/Enjoyment of Sensations,<br>6. Subjective Concentration and Reaction Ability, and<br>7. Grooming, Freshness, and Pleasant Body Perception.<br>[Endriss, L., 2023]

**Psychometric properties**	*Validity:* not investigated<br><br>*Reliability:* The test-retest reliability of the FAW is in the medium range due to its situational sensitivity (rtt between .23 and .62; Frank, 1991). However, for an instrument intended to measure habitual physical well-being, higher stability would be expected [Kolip, P. & Schmidt, B, 2006].<br><br>*Change Sensitivity:* Not investigated
**Exclusion reasons**	The FAW has not been sufficiently evaluated. Additionally, the items only reflect short-term effects, making this questionnaire unsuitable for longitudinal surveys. Moreover, many of the dimensions included lack relevance in certain contexts. Therefore, the FAW was excluded as a measurement instrument.
**MBSRQ** *Multidimensional Body-Self Relations Questionnaire*	
**Authors**	[Vossbeck-Elsebusch et al., 2014]
**Short description**	The MBSRQ is a questionnaire designed to represent body image as a multidimensional construct and evaluate aspects of appearance. It is often used in conjunction with BMI (appearance and weight).<br>Based on 34 items, the MBSRQ captures participants' attitudes towards body image, encompassing affective, cognitive, and behavioral components.
**Psychometric properties**	A validation study is available [Vossbeck-Elsebusch et al., 2014], though it does not include a comprehensive listing of psychometric criteria. Through item analysis and reliability and validity calculations, Vossbeck-Elsebusch et al. concluded that the MBSRQ demonstrates good psychometric properties. Additionally, its use on an international scale is facilitated by the availability of the instrument in English, French, and German.
**Exclusion reasons**	The MBSRQ was excluded as a questionnaire because it is frequently associated with BMI, thereby not aligning with the intended definition of physical well-being.
FBK-20 Body Image Questionnaire	
**Author**	[FBK-20; Clement und Löwe, 1996]
**Short description**	The FBK-20 is used to assess the cognitive-affective evaluation of one's own body and measures body image through 20 items representing two independent dimensions: "Negative Body Evaluation" and "Vital Body Dynamics."
**Psychometric properties**	*Validity:* The FBK-20 demonstrates high criterion validity, with both scales ranging from 0.56 to 0.65 [FBK-20; Clement and Löwe, 1996].

	Reliability: Not investigated<br><br>*Change Sensitivity:* Sensitivity varies between 65.5% and 92.6%, with an average of 81% [FBK-20; Clement and Löwe, 1996].
**Exclusion reasons**	The FBK-20 was excluded because it is primarily used for diagnosing clinically relevant body image disorders and assessing impairments in body image.
**WKV** *questionnaire on perceived physical condition (Fragebogen zur wahrgenommenen körperlichen Verfassung)*	
**Author**	[Kleinert, J., 2006]
**Short description**	The WKV measures current physical well-being during training and/or rehabilitation and is used to assess subjective adaptations.<br>Based on an adjective pool (48 items in the preliminary version: WKV-48) evaluated by experts, the final version (WKV) consists of 20 items capturing four dimensions of physical perception:<br>• Activation<br>• Fitness<br>• Flexibility<br>• Health<br>[Kleinert, J., 2006]
**Psychometric properties**	A confirmatory factor analysis did not yield satisfactory model fit for the 4-factor model. However, the reliability of the subscales and particularly the sensitivity of the scale to changes in physical condition were rated as satisfactory to very good [Kleinert, J., 2006].<br><br>All items utilized the full range of the 6-point Likert scale. Additionally, the average standard deviation of the items (SDM = 1.35) indicates sufficient variability in values across the overall sample [Kleinert, J., 2006].
**Exclusion reasons**	The WKV was excluded due to its high correlations with psychological well-being and the insufficient differentiation of the construct "Physical Well-Being" into the four defined dimensions: Activation, Fitness, Flexibility, and Health. The WKV is more suited for training management purposes.
**FKSS** *Frankfurt Body Concept Scales (Frankfurter Körperkonzeptskalen)*	

**Author**	[Deusinger, 1998]
**Short description**	The Frankfurt Body Concept Scales (FKSS) are designed to determine the individual's self-concept or perception regarding their own body.<br><br>The FKSS consist of 64 items divided into nine scales:<br>1. Health, physical condition<br>2. Body care<br>3. Physical efficiency<br>4. Body contact<br>5. Sexuality<br>6. Self-acceptance of the body<br>7. Acceptance of the body by others<br>8. Aspects of physical appearance<br>9. Dissimilatory body processes<br><br>[Deusinger, 1998]
**Psychometric properties**	There is limited information available regarding the psychometric properties of the FKSS, and its change sensitivity has not been evaluated. According to Deusinger, the FKSS can be recommended for both healthy and clinical target groups [Deusinger, 1998].
**Exclusion reasons**	The FKSS was excluded due to its extensive nature (high time commitment) and the lack of items specifically addressing physical well-being.<br>Additionally, the scales are only available in a combined book format (manual and test) that is neither freely available nor accessible online.<br>(Access: www.testzentrale.de/shop/frankfurter-koerperkonzeptskalen.html)
**FBeK** *questionnaire for assessing one's own body (Fragebogen zur Beurteilung des eigenen Körpers)*	
**Authors**	[Richter-Appelt and Strauß, 1996]
**Short description**	The FBeK is a questionnaire designed to assess subjective aspects of body experience. It consists of 52 items focusing on the areas of "physical attractiveness and self-confidence," "emphasis on physical appearance," "uncertainties and concerns regarding appearance," and "physical reactions and physical-sexual discomfort" [Richter-Appelt & Strauß, 1996].

**Psychometric properties**	Studies have already been conducted that demonstrate the concurrent and divergent validity of the FBeK in comparison to other questionnaires, showing that the FBeK is well-suited for differentiating clinical groups [Richter-Appelt & Strauß, 1996].
**Exclusion reasons**	The FBeK was excluded due to the significant time commitment required and its predominant use in clinical/psychosomatic differential and process diagnostics.<br>Furthermore, the FBeK is only available in a combined book format (manual and test), which is neither freely available nor accessible online.<br>(Access: www.testzentrale.de/shop/fragebogen-zur-beurteilung-des-eigenen-Koerpers.de)
**FEW 16** *Questionnaire for Assessing Physical Well-Being (Fragebogen zur Erfassung des körperlichen Wohlbefindens)*	
**Authors**	Kolip, P. and Schmidt, B. 1999
**Short description**	The FEW 16 is a questionnaire that, for the first time in German-speaking countries, operationalizes habitual physical well-being and defines it as an "independent state of well-being."<br>Its origins are rooted in Antonovsky's salutogenesis concept and the WHO's health definition through the operationalization of the health/illness continuum. The FEW 16 does not rely on the negative pole of the health/illness continuum [Kolip, P. & Schmidt, B, 2006].<br><br>The questionnaire assesses four dimensions of physical well-being:<br>▪ Resilience (e.g., "I am physically resilient.")<br>▪ Vitality (e.g., "I wake up feeling energized in the morning.")<br>▪ Enjoyment (e.g., "I take time to do good things for my body.")<br>▪ Inner Calm (e.g., "I feel internally balanced.").<br><br>Each scale includes four exclusively positively worded items, which are rated on a six-point Likert scale (0 = does not apply at all, 1 = hardly applies, 2 = does rather not apply, 3 = rather applies, 4 = mostly applies, 5 = fully applies) [Albani, C. et al., 2006]. A final overall score is calculated as the mean of the four scale scores.
**Psychometric properties**	Albani, C. et al. conducted a validation and standardization of the FEW 16 in 2006 with a representative German population sample.

	Validity: The psychometric properties can be considered satisfactory and indicate the validity of the instrument [Albani, C. et al., 2006].<br><br>*Internal Consistencies:* The internal consistencies of the four scales range between 0.88 ("Enjoyment") and 0.93 ("Resilience"), which are deemed good overall. The intercorrelations between the scales (0.46–0.63) were significantly exceeded in the present study (0.74–0.77) [Albani, C. et al., 2006].<br><br>*Reliability:* Not investigated<br><br>*Change Sensitivity:* Not investigated<br><br>Albani, C. et al. concluded that the FEW 16 adequately measures physical well-being through the dimensions of "Resilience," "Vitality," "Enjoyment," and "Inner Calm." However, in the non-clinical sample studied, the construct appeared to be unidimensional, suggesting that differentiation into four subscales may only provide additional information for specific research questions.<br><br>Another concern is that some items partially reflect psychological aspects of well-being (e.g., Item 6: "I feel internally balanced," Item 15: "I am calm and relaxed," or Item 16: "I am balanced") and thus fail to clearly differentiate and primarily represent physical well-being [Albani, C. et al., 2006].
**Exclusion reasons**	The FEW 16 was excluded because it predominantly represents general psychological well-being rather than subjective physical well-being.<br><br>Additionally, factor loadings are not distinct (mixed loadings on different factors), and there are overlaps with the FW-7 from the DSF.<br><br>For these reasons, the questionnaire was excluded.
**Citations**	

Literature on the Construct of Physical Well-Being:

Frank, Renate (2007). *Therapieziel Wohlbefinden. Ressourcen aktivieren in der Psychotherapie*. Springer Heidelberg. S. 135 – 143

Mommert-Jauch, P. (2022). Vierte Strategie: *Embodiment–wie mein Körper mein Denken und Fühlen beeinflusst.* In *Embodiment im Stressmanagement: Ein multimodales Kursmanual zur Förderung der Stressbewältigung* (pp. 65-92). Berlin, Heidelberg: Springer Berlin Heidelberg.

Schumacher, J., Klaiberg, A., & Brähler, E. (2003). *Diagnostik von Lebensqualität und Wohlbefinden–Eine Einführung. In Diagnostische Verfahren zu Lebensqualität und Wohlbefinden* (Vol. 2, pp. 1-329). Göttingen: Hogrefe.

World Health Organization (WHO) (1946). *Constitution*. Genf: WHO. S. 1

Literature on the available questionnaires:

Becker, P. (1989). *Der Trierer Persönlichkeitsfragebogen.* TPF. Göttingen

Bullinger, M., Kirchberger, I., Ware, J. (1995). *Der deutsche SF-36 Health Survey*. Zeitschrift für Gesundheitswissenschaften, 3: 21-36

Clement, U., Löwe, B. (1996). *The „FKB-20“, a new body image questionnaire-Literature review, description and validation.* Article in Diagnostica, December 1996, 42(4):352-376

Deusinger, I. M. (1998). *Die Frankfurter Körperkonzeptskalen (FKKS).* Hogrefe.

Kleinert, J. (2006). *Adjektivliste zur Erfassung der wahrgenommenen körperlichen Verfassung (WKV).* Zeitschrift für Sportpsychologie, 13(4), 156-164.

Strauß, B., & Richter-Appelt, H. (1996). *Fragebogen zur Beurteilung des eigenen Körpers (FBeK)*. Hogrefe Verlag für Psychologie.

Vossbeck-Elsebusch, A. N., Waldorf, M., Legenbauer, T., Bauer, A., Cordes, M., Vocks, S. (2014). *German version of the Multidimensional Body-Self-Relations Questionnaire- Appearance Scales (MBSRQ-AS): Confirmatory factor analysis and validation.* Body Image: Volume 11, Issue 3, June 2024, Pages 191-200

(https://doi.org/10.1016/j.bodyim.2014.02.002)

Literature on FEW 16:

Albani, C., Blaser, G., Geyer, M., Schmutzer, G., Hinz, A., Bailer, H., ... & Brähler, E. (2006). *Validierung und Normierung des „Fragebogen zur Erfassung des körperlichen Wohlbefindens"(FEW-16) von Kolip und Schmidt an einer repräsentativen deutschen Bevölkerungsstichprobe.* PPmP-Psychotherapie· Psychosomatik· Medizinische Psychologie, 56(03/04), 172-181.

Jahandar Lashki, D., Zelenak, C., Tahirovic, E., Trippel, T. D., Kolip, P., Busjahn, A., Düngen, H. D. (2017). *Assessment of subjective physical well-being in heart failure: Validation of the FEW16 questionnaire.* Herz, 42, 200-208.

Kolip, Petra; Schmidt, Bettina (1999). *Der Fragebogen zur Erfassung körperlichen Wohlbefindens (FEW 16): Konstruktion und erste Validierung.* Zeitschrift für Gesundheitspsychologie, 7(2):77-87.
Originalia; online veröffentlicht 01, 2006: https://doi.org/10.1026//0943-8149.7.2.77

Further Literature:

Endriss, L. (2023). *Positive Psychologie und Wohlbefinden.* In Alltägliche Parallelwelten: Flow und andere außergewöhnliche Bewusstseinszustände analysieren und bewerten (pp. 7-30). Wiesbaden: Springer Fachmedien Wiesbaden.

Frank, R. (2011). *Körperliches Wohlbefinden durch Selbstregulation verbessern. Therapieziel Wohlbefinden: Ressourcen aktivieren in der Psychotherapie*, 141-154.
